# PARylation-centric crosstalk: orchestrating immune evasion and multidrug resistance in ovarian cancer

**DOI:** 10.3389/fimmu.2026.1772562

**Published:** 2026-05-01

**Authors:** Lin Hou, Mengwen Zhang, Weihua Tong, Songling Zhang

**Affiliations:** 1 Department of Obstetrics and Gynecology, The First Hospital of Jilin University, Changchun, China; 2 Jilin Provincial Key Laboratory of Women’s Reproductive Health, Changchun, China

**Keywords:** drug resistance, immunity of cancer, ovarian cancer, PARylation, post-translational modification

## Abstract

Post-translational modifications (PTMs) play pivotal roles in ovarian cancer pathogenesis, with poly(ADP-ribosyl)ation (PARylation) serving as a key regulator of DNA repair, immune evasion, and therapeutic resistance. Beyond PARylation, diverse PTM networks—including ubiquitination, phosphorylation, acetylation, methylation, and glycosylation—orchestrate signaling cascades that shape tumor progression and immune recognition. Aberrant glycosylation of MUC16 (CA125) and immune checkpoints such as PD-L1 exemplifies how PTMs modulate the tumor immune microenvironment. This review synthesizes current evidence on the interplay between PARylation and other PTM networks in ovarian cancer, with emphasis on their roles in DNA repair, immune modulation, and drug resistance. We discuss PARP1/2-mediated regulation of cGAS/STING signaling and immune cell activity, alongside resistance mechanisms involving EHMT1/2-associated histone methylation, SPINDOC-enhanced PARylation, and ubiquitin-dependent PARP1 stabilization. Therapeutically, we evaluate combinatorial approaches pairing PARP inhibitors with ATR/CHK1 inhibition, immune checkpoint blockade, or metabolic targeting. Emerging strategies combining PARP inhibitors with PRMT, UBA1, WEE1, or MEK inhibitors are examined, alongside recent clinical trials including the GINECO study of bevacizumab, olaparib, and durvalumab. Mechanistic insights into PARP inhibitor-induced T cell DNA damage and strategies to preserve lymphocyte function are also discussed. Preclinical approaches involving nanoparticle delivery, PROTACs, and ferroptosis induction are reviewed for their potential to disrupt PARylation networks. Despite these advances, clinical translation faces substantial challenges, including patient heterogeneity, overlapping toxicities, adaptive resistance through PTM network rewiring, and the need for predictive biomarkers beyond BRCA mutation status. Current obstacles in resolving spatiotemporal PTM dynamics and cancer stem cell-specific vulnerabilities are outlined. This work aims to inform future research on targeting PARylation-associated PTM pathways to overcome ovarian cancer’s evolvable resistance.

## Graphical Abstract

**Figure:**
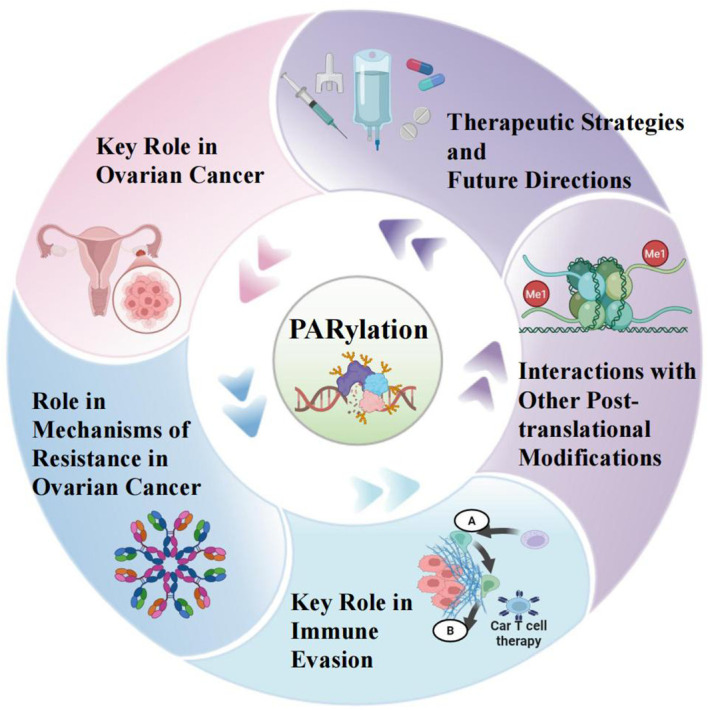
A comprehensive summary of the core roles of poly(ADP-ribosyl)ation (PARylation) and its crosstalk with other post-translational modifications (PTMs) in ovarian cancer. The diagram encapsulates four key dimensions: (1) the central function of PARylation (mediated by PARP1/2) in regulating DNA repair, immune evasion (e.g., CGAS/STING pathway suppression, PD-L1 upregulation), and tumor progression; (2) the interplay between PARylation and other PTMs (ubiquitination, phosphorylation, methylation, glycosylation, etc.) that orchestrates signaling networks driving malignant phenotypes; (3) PARylation-associated resistance mechanisms (e.g., EHMT1/2-mediated histone methylation, SPINDOC-enhanced PARylation, restored homologous recombination) limiting the efficacy of PARP inhibitors and platinum-based chemotherapy; (4) current and emerging therapeutic strategies targeting these pathways, including PARP inhibitors (olaparib/niraparib/ruc parib), combination regimens (PARP+ATR/CHK1/WE E1 inhibitors, immune checkpoint blockade), and novel modalities (nanoparticle delivery, PROTACS, ferroptosis induction). This overview highlights the multifaceted role of PARylation as a central orchestrator of ovarian cancer pathogenesis and provides a framework for understanding PTM-targeted therapeutic development

## 1 Introduction

Post-translational modifications (PTMs) are pivotal in tumor development, immune regulation and drug resistance (1–3). Key reversible modifications including phosphorylation, acetylation and PARylation regulate essential biological processes like cellular signal transduction, gene expression, and the cell cycle (1, 4–7). Poly(ADP-ribosyl)ation (PARylation), as a significant PTM, influences protein stability and activity, thereby participating in various biological processes including DNA repair, apoptosis, and immune responses (8–10). In ovarian cancer, aberrant expression of post-translational modifications is closely linked to tumor progression (11–14). Emerging evidence indicates markedly elevated PARylation levels in PARylation levels within ovarian cancer cells, which is associated with the tumor immune micro-environment and drug resistance (15–17).

Beyond PARylation, a diverse array of PTMs—including ubiquitination, phosphorylation, acetylation, methylation, and glycosylation—orchestrate complex signaling networks that shape ovarian cancer pathogenesis (13, 18–22). These modifications not only regulate cell-autonomous processes such as proliferation and DNA repair but also modulate intercellular communication within the tumor microenvironment, influencing immune cell recruitment, activation, and exhaustion. Understanding how these PTM networks interconnect is essential for developing effective therapeutic strategies.

The clinical efficacy of PARP inhibitors in BRCA-deficient ovarian cancer is counterbalanced by rising resistance in ovarian cancer patients with BRCA deficiencies, the development of resistance remains a major clinical hurdle (23–25). Research on ovarian cancer-specific PTM profiles reveals that PARylation plays a crucial role in regulating tumor cell survival, proliferation, and response to chemotherapy (26–28). PARylation additionally shapes the tumor microenvironment through direct interactions with malignant and immune cells in shaping the tumor microenvironment through its interaction with tumor and immune cells (29–33). However, the dynamic interplay between PARylation and other PTMs—such as ubiquitination-dependent PARP1 stabilization, SUMOylation-mediated DNA repair, or glycosylation-driven immune evasion—creates adaptive resistance mechanisms that limit the durability of therapeutic responses (34–38).

The emergence of resistance to PARP inhibitors has spurred intensive investigation into combination strategies that co-target parallel PTM pathways. Preclinical and early-phase clinical studies are now exploring regimens pairing PARP inhibitors with ATR/CHK1 inhibitors, immune checkpoint blockade, or novel agents targeting PRMTs, UBA1, WEE1, or MEK—each designed to exploit specific vulnerabilities in resistant tumor cells (39–47). These efforts are increasingly guided by biomarker-driven patient selection, recognizing that ovarian cancer encompasses multiple histotypes with distinct molecular landscapes and variable immune infiltrate compositions.

Despite these advances, translating combination regimens into clinical practice faces substantial challenges. Overlapping toxicities, optimal dosing schedules, and the inevitable emergence of acquired resistance through PTM network rewiring remain unresolved. Moreover, reliable predictive biomarkers beyond BRCA mutation status are urgently needed to identify patients most likely to benefit from specific PTM-targeted therapies. An in-depth exploration of the mechanisms underlying PTMs in ovarian cancer, particularly the interplay between PARylation and other modifications, can unlock new clinical therapeutic strategies. This review synthesizes current evidence on PARylation-centric PTM crosstalk in ovarian cancer, with emphasis on its impact on DNA repair, immune evasion, and therapeutic resistance. We discuss emerging combination therapies targeting parallel PTM pathways, summarize ongoing clinical trials, and outline key challenges and future directions for translating these insights into durable clinical benefits. Understanding PTM multifunctionality, particularly in immune evasion and treatment resistance, should guide subsequent research prioritization.

## 2 PARylation and technology development

### 2.1 PARylation and PARPs

PARylation, which is mediated by poly(ADP-ribose) polymerases (PARPs), is a key PTM to DNA injury repair and genome stabilization (48). PARP1, a major member of PARP family, identifies DNA injury, catalyzes PAR synthesis and promotes repair (49). PARP1 has been associated with the development of tumor cells in ovarian cancer, particularly in those with BRCA1/2 mutations for which PARP inhibitors have exhibited a positive response (50–54). Furthermore, a variety of researches have investigated the role of PARylation in the regulation of cell processes. For example, PARylation has been shown to control the dynamics of stress granules and the phase separation of RNA-binding proteins that are important in the neurodegenerative disorders (55). PARylation also associates with the regulation of histone methyltransferase activity and chromatin binding, which in turn affects gene activation and DNA damage recovery (56).

PARP1 catalyzes the DNA damage site and then the synthesis of the PAR chain with NAD + as the substrate (57–59). This process not only recruits DNA repair proteins but also affects the fate of the cell by inducing apoptosis and regulating the expression of the gene (60–63). In the case of ovarian cancer, the activity of PARP1 plays a key role in drug resistance (28, 64–67). For example, PARP1 interacts with major DNA damage reaction factors such as BRCA1 or ATM, which might have a considerable impact on PARPi sensitivity of ovarian cancer cells (68, 69).

Most PARP family members catalyze mono(ADP-ribosyl)ation (MARylation), whereas others like PARP2 and tankyrases (PARP5A/PARP5B) characterize multiple PARylation functions (70–81). Compared with PARP1 recruited by DNA damage-mimicking oligonucleotides, PARP2 which is activated by 5′-phosphorylated DNA breaks, shares and overlaps some of the auto-modifying function to a lesser extent (72, 82–85). Although tankyrases are not nuclear proteins, they regulate various cellular processes as diverse as telomere maintaining, mitotic processing, DNA damage response, Wnt and LKB1/AMPK signaling (86–90). For over half a century, the PARP family and PARylation have been intensively studied. As biomolecular mechanisms are further explored and technologies refined, additional aspects of PARylation are being clarified, though many questions persist (91).

### 2.2 Progresses of detection technology and research strategies

Over the past twenty years, transformative advances in PARylation detection technologies have enabled multidimensional interrogation of this dynamic modification (86, 92–116). The synergy of biological sensors with advanced analytical platforms now overcomes historical limitations in detecting low-abundance PARylation events. A key innovation couples bimolecular PARylation biosensors to genetic screening—exploiting the PAR-binding zinc finger (PBZ) domain as a molecular scaffold—to achieve spatiotemporal mapping in living cells. The systematic integration across protein-coding genes revealed previously unidentified targets exemplified by CTIF (CBP80/CBP20-dependent translation initiation factor), whose tankyrase-mediated centrosomal PARylation was confirmed through orthogonal validation (86).

Genetically encoded sensors further permit real-time tracking *in vitro* and *in vivo*. Thomas et al.’s pARS FRET sensor capitalizes on PAR chain oligomerization to quantify PARP1 dynamics under physiological DNA damage, unexpectedly enabling *in situ* assessment of inhibitor potency in living systems (114). Concurrently, background-suppressed AIE sensors leverage electrostatic switching: PARP1-triggered aggregation of cationic TPE-Py on nascent PAR polymers generates ratiometric fluorescence, achieving interference-resistant monitoring in cancer models (93).

Electrochemical strategies now expand this arsenal. Guanidine-functionalized peptides template CuNPs while autonomously labeling PAR, enhancing selectivity for voltammetric quantification (109). Polycationic PFP polymers convert PAR binding into photocurrent signals for tracking in breast/ovarian cancers (110), whereas hyperbranched PAR polymers induce quantifiable steric gating in nanochannels to profile inhibitor efficacy (98). Methylene blue adsorption biosensors attain attomolar sensitivity through PAR-dependent electrochemical shifts (116). Through structure-guided design, the researchers developed PARP1 inhibitors exhibiting >60-fold selectivity over PARP2. Y49 showed considerable suppression of BRCA1-deficient tumor growth in cell line-derived xenograft models while maintaining favorable tolerability (117). Collectively, these technological advances decoded PARylation’s biological complexity and catalyzed new therapeutic paradigms, advancing beyond basic research toward clinical translation.

## 3 PARylation and other PTMs in cancer immunity

### 3.1 Mechanisms of PARylation in cancer immunity

PARylation influences cancer immunity through DNA repair mechanisms and by modulating immune cell activities and cytokine production. PARP1 and PARP2 inhibit antitumor responses via their regulation of immune checkpoint expression and cGAS/STING signaling (118). In the cGAS-STING pathway, these enzymes block activation of IRF-3 and NF-κB, leading to reduced production of type I interferons and pro-inflammatory cytokines. Such cytokines typically attract conventional dendritic cells, CD8+ T cells, and NK cells to tumor sites (119–122). PARP1 modulates immune responses by targeting macrophages and T cells, which affects tumor development (33, 123–125). In macrophages, PARP1 activity drives polarization toward the pro-inflammatory M1 phenotype, which is important for initiating antitumor immunity. Macrophages shift between M1 and anti-inflammatory M2 states; PARP1 promotes M1 dominance. Although this effect supports immune activation, excessive PARP1 activity may cause chronic inflammation and foster a tumor-promoting milieu (123), suggesting that while beneficial in moderation, its overstimulation requires careful validation in inflammatory contexts. In T cells, this enzyme controls cytokine expression and signaling molecules critical for activation and proliferation. PARP1’s function in T cells displays complexity: it enhances immune responses yet can suppress them under specific conditions, particularly in cancer where it either aids antitumor immunity or enables tumor immune evasion (126, 127). PARP1 also interacts with key immune regulatory pathways, such as STAT3, which governs PD-L1 expression used by tumors to evade detection. Through this interaction, PARP1 shapes cancer cell immune escape mechanisms, underlining its potential as an immunotherapy target (128, 129). The considerable immunomodulatory impact of PARylation supports the rationale for exploring PARP1-targeted strategies.

### 3.2 Role of other PTMs in immune response to cancer

Histone methylation, acetylation, and ubiquitination serve as established epigenetic markers, while phosphorylation, lactylation, glycosylation, butyrylation, and propionylation contribute additional regulatory layers (**Table 1**). These alterations directly impact chromatin architecture and transcriptional activity, which modulates immune checkpoint expression and function. Such changes affect core cellular pathways relevant to cancer development and immune checkpoint blockade efficacy (139, 140). Dysregulated histone modifications frequently promote tumor proliferation, invasive potential, apoptotic resistance, and stem-like properties. Within triple-negative breast cancer models, OTUD4 governs CD73 expression through post-translational regulation. Disrupting the OTUD4/CD73 signaling axis reduces immunosuppression, though clinical validation remains necessary (141). Ubiquitin signaling exerts broad control over cellular homeostasis; its dysregulation features prominently in cancer pathogenesis (142). This modification also shapes cancer immunity by altering immune cell behavior and immune-related molecules. Ubiquitylation events may degrade proteins central to immune checkpoint control, tipping the balance between anti-tumor and pro-tumor immunity.

**Table 1:** Types, mechanisms, and therapeutic strategies of post-translational modifications in ovarian cancer.

PTM type	Mechanism of action	Relation to ovarian cancer immunity	Mechanisms of resistance	Related therapeutic strategies	References
Phosphorylation	Addition of phosphate groups to proteins, regulating signal transduction, gene expression, and the cell cycle.	Phosphorylation regulates immune checkpoint expression and immune cell activation, affecting immune surveillance in tumors.	Altered phosphorylation of key signaling proteins (e.g., PI3K/AKT/mTOR pathway) leads to survival and chemotherapy resistance.	Targeting phosphorylation pathways (e.g., PI3K inhibitors) to restore sensitivity to chemotherapy and immunotherapy.	(11, 130, 131)
Ubiquitination	The attachment of ubiquitin to proteins, leading to degradation or modification of protein activity.	Ubiquitination regulates immune cell activity and cytokine production, influencing tumor immune evasion mechanisms.	Dysregulated ubiquitination can lead to protein stabilization, enhancing tumor survival and resistance to apoptosis.	Inhibitors targeting deubiquitinases (e.g., USP22) to reverse resistance and restore chemotherapy efficacy.	(12, 38, 132)
Acetylation	Addition of acetyl groups to lysine residues, influencing gene expression, chromatin structure, and protein function.	Modifies histones and immune-related molecules, influencing immune checkpoint regulation and tumor growth.	Altered acetylation of histones can result in tumor resistance to chemotherapy by enhancing chromatin accessibility and gene activation.	Histone deacetylase (HDAC) inhibitors to reverse chemoresistance by modifying chromatin structure and gene expression.	(133, 134)
Methylation	Addition of methyl groups to DNA or histones, influencing gene expression and chromatin remodeling.	Methylation of immune-related genes can silence tumor suppressors, contributing to immune evasion and resistance.	DNA and histone methylation lead to silencing of tumor suppressor genes, promoting resistance to chemotherapy and targeted therapy.	DNA methyltransferase inhibitors (e.g., 5-Aza-2-deoxycytidine) to reverse epigenetic changes and resensitize tumors to treatment.	(20, 135, 136)
SUMOylation	Attachment of SUMO (Small Ubiquitin-like Modifier) proteins, altering protein stability and activity.	SUMOylation regulates immune checkpoint proteins and cytokine production, impacting immune responses in the tumor microenvironment.	SUMOylation of key proteins can enhance immune evasion and resistance to chemotherapy by stabilizing survival pathways.	Targeting SUMOylation pathways to enhance immune response and sensitize tumors to chemotherapy and immunotherapy.	(36, 37, 137)
PARylation	Addition of poly(ADP-ribose) chains to proteins, involved in DNA repair and cellular stress response.	PARylation regulates immune cell functions (e.g., macrophages, T cells) and immune checkpoint activation, influencing immune evasion.	Overactive PARylation can promote DNA repair and survival, leading to resistance to PARP inhibitors and chemotherapy.	PARP inhibitors and combination therapies with immune checkpoint inhibitors to overcome resistance in PARylation-driven cancers.	(26, 28, 138)

Protein lactylation, identified recently, represents another notable regulator in cancer systems. Lactylation modulates both transcriptional programs and protein activities, supporting metabolic adaptation in tumors and remodeling the immune microenvironment (143). Post-translational modifications additionally determine the immunogenicity of tumor-associated antigens. Acetylation, citrullination, and phosphorylation often enhance antigen presentation, strengthening anti-tumor immune responses across diverse malignancies (144). These mechanisms position PTMs as potential targets for cancer vaccine design.

Altered glycosylation patterns are a hallmark of malignancy, affecting protein stability, cell adhesion, and signaling (130, 145, 146). In ovarian cancer, the most prominent example is the massive glycosylation of MUC16 (CA125), a clinically used serum biomarker (133). Specifically, sialylated glycan structures on MUC16 engage Siglec receptors on immune cells, such as Siglec-9 on macrophages and NK cells, transmitting inhibitory signals that promote immune evasion (135, 147). Similarly, aberrant glycosylation of MUC1, another mucin overexpressed in ovarian cancer, disrupts cell–cell adhesion and exposes tumor-associated antigens, yet also creates immunosuppressive glycan ligands that bind to galectins on T cells, dampening antitumor responses (148, 149). Beyond mucins, glycosylation directly influences key immune checkpoint molecules. For instance, N-linked glycosylation of PD-L1 at specific asparagine residues stabilizes the protein and prevents its ubiquitination-mediated degradation, thereby sustaining PD-1/PD-L1 inhibitory signaling in the tumor microenvironment (150–152). Moreover, alterations in the glycosylation of the T cell receptor or MHC molecules can affect antigen presentation and T cell activation, further shaping the immune landscape (153, 154).

Reactive nitrogen species generate further PTMs within tumor microenvironments. RNS-dependent modifications establish chemical barriers that hinder effector T cell infiltration and function, accelerating immune escape (155). Counteracting such modifications might yield new biomarkers or refine therapeutic approaches. Hepatocellular carcinoma exemplifies cancer-type-specific PTM roles, where these alterations bidirectionally regulate immune checkpoint molecules and immune cell activity, ultimately shaping tumor immune evasion (156).

Non-canonical modifications like O-GlcNAcylation and glutathionylation also disrupt cellular signaling in cancer. Their diagnostic and therapeutic potential warrants deeper exploration (157). Specific protein 1 activity further illustrates PTM importance: phosphorylation, ubiquitination, and acetylation collectively determine Sp1 stability, DNA-binding capacity, and transcriptional output—processes important for cancer progression (158). Collectively, PTMs orchestrate multifaceted immune responses by targeting checkpoints, tumor antigens, and microenvironmental factors. Deciphering these networks may lead to improved immunotherapies.

### 3.3 Interactions between PARylation and other PTMs in cancer cells

PARylation dynamically engages with key post-translational modifications including ubiquitination and SUMOylation, collectively steering cellular functions that drive tumor development. This molecular crosstalk substantially impacts cancer progression and therapeutic efficacy. A particularly consequential relationship exists between PARylation and ubiquitin signaling. PARylation frequently marks proteins for subsequent ubiquitylation and proteasomal destruction through PAR-dependent ubiquitination (PARdU). The tankyrase ADP-ribosyltransferase and E3 ligase RNF146 execute this pathway, coordinating DNA repair alongside other essential processes (159). Disrupting PARdU has therapeutic potential since it governs tumorigenic pathways, immune evasion, and cell death mechanisms.

Breast cancer exemplifies important PARylation-SUMOylation interplay where SUMOylation cooperates with ubiquitination to control disease mechanisms. Such coordination occurs at precise protein sites, directly influencing oncogenesis. Deciphering these interactions may reveal new breast cancer treatments, though validation in physiological contexts remains essential (137). PARylation further modulates the p53 tumor suppressor, with its C-terminal domain serving as a central regulatory platform. ADP-ribosylation here alters p53’s DNA-binding characteristics and functional outputs, thereby reshaping its interactome and associated cellular activities (160).

Ubiquitin signaling reciprocally regulates PARP1 activity. The HECT-type E3 ligase SMURF2 binds PARP1, enhancing its PARylation function and stabilizing expression levels (161). During DNA damage response, BRCA1 PARylation appears indispensable. Its inhibition causes excessive DNA end resection, compromising homologous recombination repair—a process with direct implications for PARP inhibitor therapies (162). A USP10-mediated deubiquitination-PARylation feedback loop also amplifies DNA repair capacity. Targeting this circuit could potentially augment PARP inhibitor efficacy in breast cancer, though clinical translation requires further investigation (38). PARylation networks show profound complexity in cancer systems, intersecting with diverse PTMs to influence therapeutic vulnerabilities and tumor biology.

## 4 PARylation and other PTMs-related mechanisms of ovarian cancer resistance

### 4.1 PARylation in the context of ovarian cancer

PARylation contributes to pathological mechanisms in ovarian cancer. Recent studies reveal EHMT1 and EHMT2 (GLP/G9A) histone methyltransferases sustain PARP inhibitor resistance within high-grade serous ovarian carcinoma models. Resistant high-grade serous ovarian cancer (HGSOC) cells show globally elevated H3K9me2 marks alongside EHMT1/2 overexpression. Disrupting EHMT1/2 function through genetic knockdown or pharmacological inhibitors restores PARPi sensitivity in these cells, suggesting PARylation-associated epigenetic alterations may facilitate therapeutic escape (163). SPINDOC interacts with PARP1 to potentiate PARylation events. Depletion of SPINDOC diminishes cellular PARylation levels and heightens susceptibility to ionizing radiation-induced DNA lesions. This SPINDOC-PARylation axis is important for DNA damage management and may influence ovarian cancer pathogenesis alongside treatment resistance (164).

PARylation underpins DNA repair pathways exploited by ovarian malignancies. PARP inhibitors target tumors bearing BRCA1/2 mutations or homologous recombination defects, leveraging cancer cell dependence on PARP-mediated repair to induce genomic collapse (165, 166). Yet PARPi resistance emerges via mechanisms like restored HR capacity or enhanced drug efflux, which complicates clinical management in certain cases (167, 168). PARylation also intersects with metabolic stress responses. CHK1 inhibition via prexasertib elevates PARylation while depleting NAD+ pools in ovarian cancer cells, indicating PARP1/2 hyperactivation. Combined PARG blockade to inhibit dePARylation exacerbates replication stress under CHK1 suppression. This dual approach disrupts metabolic-DNA repair crosstalk, provoking replication catastrophe and mitotic failure (138). Although this strategy has potential against chemoresistance, its efficacy requires validation across diverse models (138).

Oxidative stress modulation constitutes another PARylation-linked pathway. PARP inhibition may exert antitumor effects partly through reactive oxygen species accumulation (169). Clinically, elevated ADP-ribosylation correlates with improved platinum and PARPi responses, suggesting possible utility as a predictive biomarker (170). The tumor microenvironment further integrates PARylation signaling. Immune modulation via PARP activity presents opportunities for combination regimens targeting both DNA repair and immunogenic pathways (17).

### 4.2 Role of PTMs in the therapeutic resistance of ovarian cancer

PTMs including ubiquitination, phosphorylation, acetylation, and methylation alter protein function, stability, localization, and binding interactions. These changes drive cancer cell survival and drug resistance. Ubiquitin attachment to substrate proteins regulates degradation and processes like DNA repair and apoptosis. In epithelial ovarian cancer (EOC), samples and cell lines exhibit upregulated ubiquitin specific protease 22 (USP22), linking high USP22 levels to advanced FIGO stage, lymph node spread, and poor outcomes. Depleting USP22 reduces cell growth *in vitro* and tumor expansion *in vivo*, as inhibition triggers G1 phase cell cycle arrest through synergy with oncogenic transforming growth factor-β1 (TGFB1) (132). Deubiquitinases (DUBs) modulate resistance by affecting protein stability in DNA damage repair, contributing to platinum therapy resistance where DNA damage kills cancer cells (171). Beyond ubiquitination, pharmacological inhibition of CDK7 selectively curbs cell proliferation in both primary cells and established lines following the characterization of CDK7 targets in EOC. Phosphorylation, involving phosphate group addition and regulating the PI3K/AKT/mTOR pathway, commonly activated in ovarian cancer and depends on phosphorylation events for roles in proliferation, survival, and drug evasion. Specific inhibitors targeting this pathway may overcome chemotherapy resistance, though clinical validation remains ongoing (131, 172). Acetylation, especially of histones, influences chromatin structure and gene activity. Altered histone acetylation correlates with chemotherapy resistance in this malignancy. Therapies aimed at histone acetylation seek to reverse resistance and enhance treatment efficacy (134).

**Figure 1:**
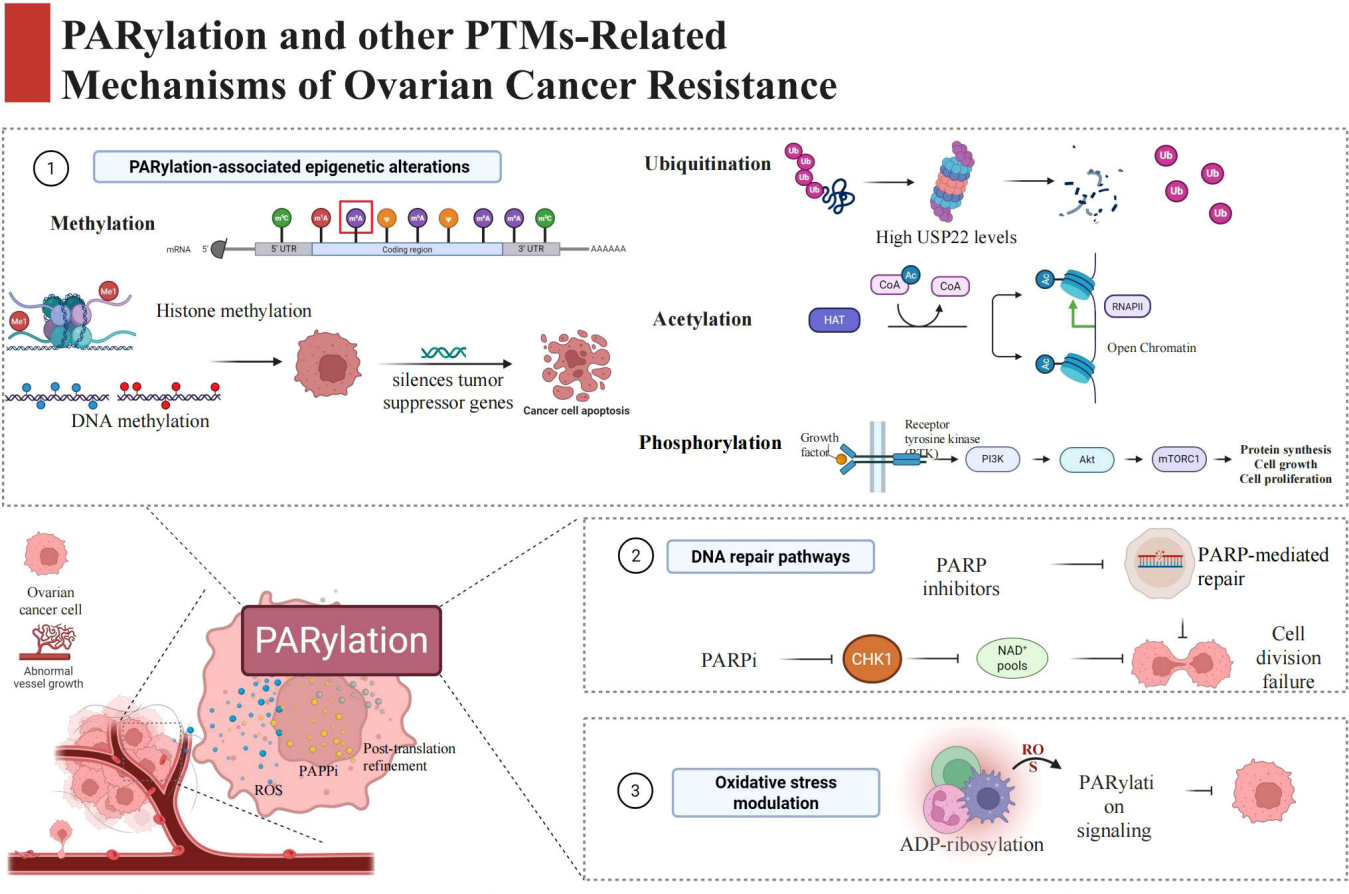
PARylation-centered PTM regulatory network orchestrates therapeutic resistance in ovarian cancer, which is divided into three pivotal mechanistic modules. (1) PARylation-associated epigenetic alterations: including histone methylation and DNA methylation that silence tumor suppressor genes to inhibit cancer cell apoptosis, m6A RNA methylation modification, alongside other key PTMs (ubiquitination, acetylation, phosphorylation) that drive resistance via regulating protein stability, chromatin state, and oncogenic signaling cascades (e.g., PI3K/Akt/mTOR pathway). (2) DNA repair pathways: PARylation-mediated DNA damage repair is the core target of PARP inhibitors (PARPI), while PARPI combined with CHK1 inhibition disrupts DNA repair capacity by depleting NAD+ pools, ultimately leading to cell division failure and therapeutic resistance. (3) Oxidative stress modulation: ADP-ribosylation regulates PARylation signaling via reactive oxygen species (ROS), which further modulates the oxidative stress response of ovarian cancer cells and contributes to acquired resistance. The central schematic highlights PARylation as the core hub integrating upstream tumor microenvironmental stimuli and PTM network rewiring to drive ovarian cancer therapeutic resistance.

Methylation, covering DNA and histone changes, also underpins resistance. For example, METTL3-mediated maturation of miR-126-5p via m6A modification of pri-miR-126-5p promotes ovarian cancer progression through PTEN-mediated PI3K/Akt/mTOR signaling (136). Elevated miR-126-5p boosts proliferation, migration, and invasion in samples and lines, and inhibits apoptosis by binding PTEN. DNA methylation silences tumor suppressor genes to foster resistance. Histone methylation alters gene expression patterns that support survival mechanisms (173). Epithelial cell adhesion molecule (EpCAM) expression in ovarian carcinoma ties to DNA methylation and histone adjustments. Treatment with 5-Aza-2-deoxycytidine (5-AZAC) induces EpCAM in negative cells, and ten transcription factors associate with the epcam gene only in expressing cells. Methylation of an Sp1 probe blocks nuclear extract protein binding, highlighting epigenetic control in EpCAM overexpression (174). CD133, a cancer stem cell marker, displays epigenetic regulation in lines. DAC treatment raises CD133 mRNA and protein levels, whereas Trichostatin A (TSA) lowers mRNA in most cases. CD133 P2 promoter methylation inversely correlates with expression (175). Integrative genomic analysis reveals epigenetic marks potentially mediating genetic risk for EOC. Causal Inference Test (CIT) identified 17 CpG/SNP pairs as methylation-mediated genotype-EOC risk links (176). Overall, PTMs appear fundamental to therapeutic resistance development in ovarian cancer (**Figure 1**). Investigating these changes and their pathway impacts provides information for targeting. Focusing on specific PTMs could yield strategies to counter drug resistance and boost existing treatment effectiveness, although further studies must confirm these approaches (177, 178).

## 5 Therapeutic strategies targeting PARylation and PTMs in ovarian cancer

### 5.1 Current therapeutic approaches targeting PARylation in ovarian cancer

PARP inhibitors are central to current ovarian cancer therapeutics, leveraging synthetic lethality to target DNA repair defects. Agents like olaparib, niraparib, and rucaparib gained approval for recurrent disease, especially in BRCA-mutated patients, by capitalizing on homologous recombination deficiencies (166, 179). In women with BRCA1/2 mutations who respond to first-line chemotherapy and receive maintenance olaparib, maintenance PARP inhibitor therapy has led to a considerable prolongation of progression-free survival compared to placebo (60% vs 27%) (166). Combining these inhibitors with other agents emerges as a strategy to boost efficacy and counter resistance. Pairing PARP inhibitors with immune checkpoint blockade generated enhanced anti-tumor immune responses in preclinical models, though human data requires further validation (180). Concurrent targeting of PARP and PI3K/AKT/mTOR pathways addresses resistance in platinum-resistant cases, where pathway crosstalk undermines monotherapy (181).

Dual inhibition approaches show potential. Coordinated PARP and Aurora kinase A suppression synergistically reduced tumor growth in preclinical ovarian cancer models, extending survival (182). Disrupting the cGAS-TBK1-IRF3 signaling axis presents another route to circumvent PARP inhibitor resistance, as this pathway may fosters inflammation-mediated evasion (183). Separately, inhibiting poly (ADP-ribose) glycohydrolase (PARG) alongside CHK1 blockade induces lethal replication stress and metabolic collapse in chemoresistant cells (138). This underscores how targeting PARylation dynamics may disrupt cancer metabolism to potentiate therapies. Integrating PARP inhibitors with complementary modalities offers pathways for improving ovarian cancer management. Active clinical exploration continues to define optimal combinations to broaden therapeutic options for patients, while acknowledging that long-term efficacy data remains maturing (184, 185).

Beyond monotherapy, the clinical landscape of PARP inhibitors is rapidly evolving through combination strategies. A growing number of clinical trials are evaluating PARP inhibitor-based combinations with targeted agents, immunotherapies, and anti-angiogenic drugs, aiming to broaden efficacy and circumvent resistance (186–192). These efforts are summarized in **Table 2** and discussed in detail in the following sections.

**Table 2:** Clinical trials of PARP inhibitor combinations targeting PTM networks.

Year	Cancer type	Targeted PTM network	Drug combination	Study population	Key findings	References
2026	Ovarian	VEGFR2-STAT3 phosphorylation network	Fuzuloparib + Apatinib	First-line maintenance for newly diagnosed advanced ovarian cancer (FZOCUS-1, Phase III)	In HR-proficient subgroup, PFS showed improvement trend with combination (16.6 vs 11.0 months); no benefit in HRD population.	(186)
2026	Prostate	Androgen receptor PTM network (phosphorylation, acetylation, ubiquitination)	Talazoparib + Enzalutamide	Metastatic castration-resistant prostate cancer, unselected for HRR status (TALAPRO-2, Phase III)	Final OS: median OS 45.8 vs 37.0 months (HR 0.796, p=0.0155); 20.4% reduction in death risk.	(191)
2023	Ovarian	DNA damage response phosphorylation network (ATR-CHK1 signaling)	Olaparib + Ceralasertib	HR-deficient ovarian cancer with prior PARP inhibitor benefit and subsequent progression (CAPRI trial, Phase II)	ORR 50% (6/12 PR); median 8 cycles; CBR 86%.	(190)
2021	Ovarian	DNA damage response phosphorylation network (ATR inhibition in platinum-resistant setting)	Olaparib + Ceralasertib	Recurrent, platinum-resistant epithelial ovarian cancer (CAPRI trial, platinum-resistant cohort, Phase II)	No objective responses observed; BRCA1-mutant patients showed activity signal; PFS 4.2 months.	(189)
2021	Multiple (including ovarian)	DNA damage response phosphorylation network (ATR inhibition in DDR-altered tumors)	Olaparib + Ceralasertib	Advanced solid tumors harboring DNA damage response alterations, including PARPi-resistant HGSOC (Olaparib Combinations basket trial, Phase I/II)	In PARPi-resistant HGSOC (n=7): CBR 86% (1 PR, 5 SD).	(192)
2019	Ovarian	VEGF signaling phosphorylation network + hypoxia-induced PTMs	Olaparib + Bevacizumab	First-line maintenance for newly diagnosed advanced ovarian cancer (PAOLA-1, Phase III)	Significant PFS improvement in HRD-positive patients (HR 0.33).	(188)

### 5.2 Novel therapies addressing PTM-related resistance mechanisms

Targeting PTM-associated resistance mechanisms create opportunities for innovative ovarian cancer therapies. Disrupting the ATR/CHK1 pathway compromises DNA damage response and cell cycle control, sensitizing cells to PARP inhibitors where restored homologous recombination or stabilized replication forks cause resistance (69, 193, 194). MicroRNA (miRNA) networks actively regulate chemoresistance in epithelial ovarian cancer. Modulating specific miRNAs alters expression of resistance-associated genes, potentially restoring chemosensitivity (195).

The tumor microenvironment (TME) plays a key role in enabling resistance through metabolic rewiring and crosstalk with cancer-associated fibroblasts, immune infiltrates, and vasculature. Targeting TME components like JNK/p38 MAPK signaling reverses chemoresistance in preclinical models, though clinical translation requires optimization (196, 197). Nanoparticle systems like pH-responsive liposomes and antibody-drug conjugates circumvent resistance by overcoming drug efflux pumps and apoptosis defects while enhancing tumor specificity (198).

Inducing ferroptosis, an iron-dependent cell death distinct from apoptosis, synergizes with platinum therapies. Glutathione peroxidase 4 (GPX4) suppression triggers lethal lipid peroxidation in resistant cells, yet tissue toxicity concerns necessitate targeted approaches (199). Collectively, countering PTM-driven resistance requires integrated strategies: intercepting DNA repair cascades, reprogramming the TME, deploying advanced drug carriers, and activating alternative cell death pathways. These approaches require rigorous validation but may ultimately overcome ovarian cancer’s adaptive resilience.

Beyond these broader strategies, a growing number of specific molecular targets are being explored in combination with PARP inhibition. Recent preclinical and early-phase clinical studies have expanded the repertoire of combination partners for PARP inhibitors. For instance, protein arginine methyltransferase (PRMT) inhibitors have shown synthetic lethality with PARP inhibition in homologous recombination-proficient ovarian cancer models, likely through epigenetic dysregulation of DNA repair genes (46, 47). Similarly, targeting the ubiquitin-activating enzyme UBA1 has been proposed to enhance PARP inhibitor sensitivity by disrupting ubiquitin-dependent DNA damage signaling (43, 200).

The WEE1 kinase inhibitor adavosertib, which abrogates the G2/M checkpoint, has demonstrated synergistic activity with PARP inhibitors in multiple preclinical studies and is currently under evaluation in phase II trials for recurrent ovarian cancer (40, 201–203). Another promising avenue involves combining PARP inhibitors with MEK inhibitors, particularly in RAS-mutated or serous ovarian carcinomas, where MAPK pathway activation drives resistance to DNA damage-induced apoptosis (42, 204).

Beyond direct tumor cell killing, emerging evidence suggests that PARP inhibitor-based combinations can modulate the immune microenvironment. A recent phase II trial by the GINECO group evaluated the triplet combination of bevacizumab, olaparib, and durvalumab in relapsed ovarian cancer, reporting manageable safety and encouraging activity in biomarker-selected populations, particularly those with BRCA mutations or homologous recombination deficiency (205). Conversely, mechanistic studies have highlighted that PARP inhibitor-induced DNA damage in T cells may limit antitumor immunity (124, 206, 207). How to resolve this contradiction may require further exploration of innovative strategies—for example, through sequential therapy or dose adjustment, to preserve antitumor efficacy while minimizing the impact on T cells (208).

### 5.3 Combination therapies for overcoming ovarian cancer resistance

Overcoming therapeutic resistance in ovarian cancer—particularly to platinum agents and PARP inhibitors—poses persistent clinical challenges. Integrating PARylation targeting with complementary PTM interventions represents a potential approach. Combining PARP and ATR inhibitors enhances replication fork collapse and double-strand breaks, triggering apoptosis in resistant models where restored DNA repair mechanisms compromise monotherapy efficacy (209). Mifepristone-olaparib co-targeting reduces polyploid giant cancer cell (PGCC)-driven tumor growth in patient-derived xenografts, supporting PGCC targeting as a viable approach to overcome PARPi resistance (210).

Metabolic and DNA repair crosstalk offers intervention points. Concurrent CHK1 and PARG inhibition induces lethal replication/metabolic stress in chemoresistant cells, though optimal dosing regimens require refinement (138). PARP inhibition elevates tumor immunogenicity in subsets of patients, potentially synergizing with immune checkpoint inhibitors to improve progression-free survival clinically, despite variable patient responses (211). Cancer stem cells (CSCs) contribute substantially to resistance and metastasis. Depleting CSC populations through surface marker-specific therapies may improve outcomes, though isolation heterogeneity complicates targeting (212). PLGA nanoparticle systems overcome cisplatin resistance by enhancing intracellular drug accumulation while bypassing efflux pumps, yet biodistribution challenges remain (213).

**Figure 2:**
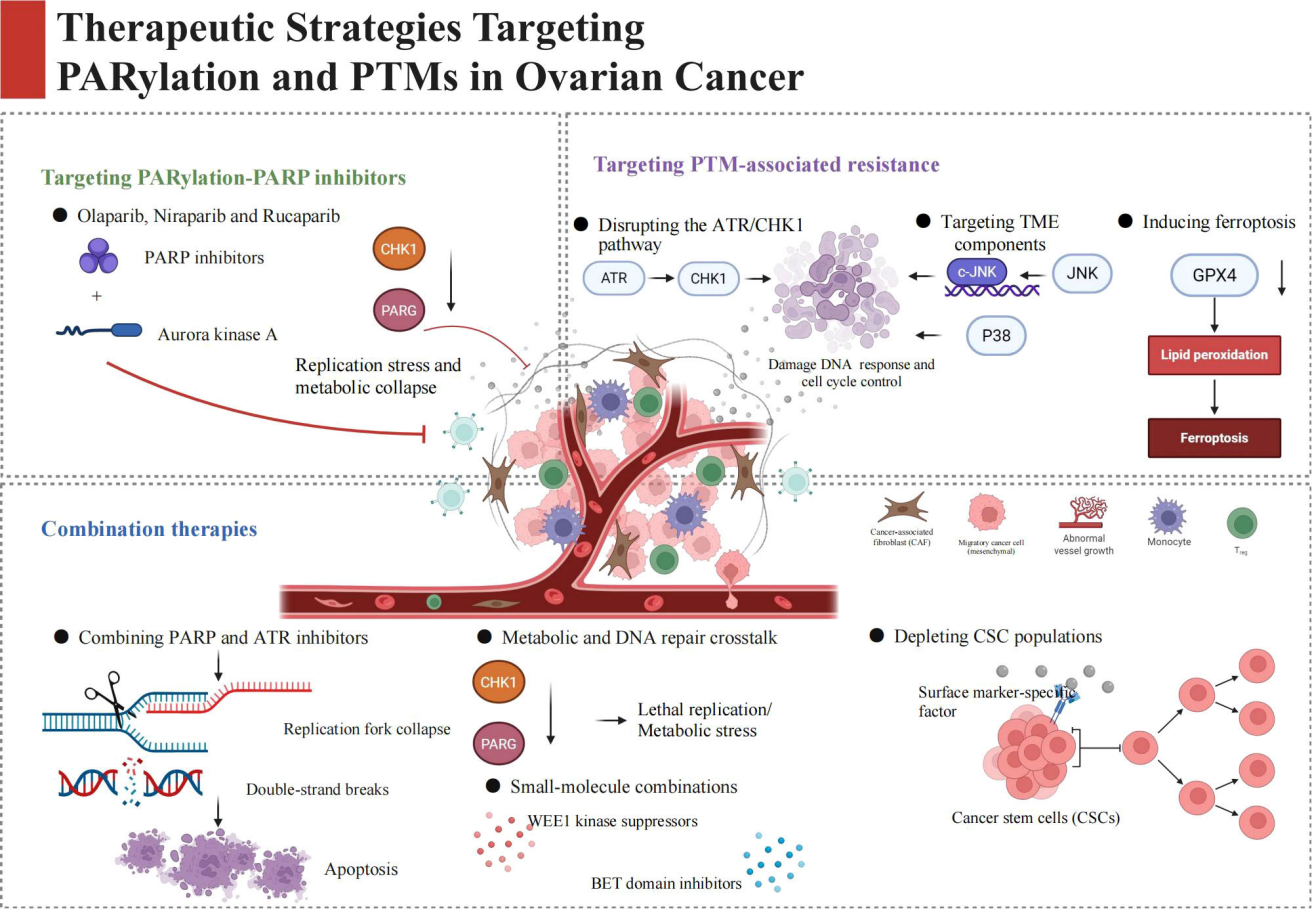
This is a comprehensive overview of PARylation-centered therapeutic regimens for ovarian cancer, with the tumor microenvironment as the core pathological scenario, divided into three key therapeutic dimensions. (1) Targeting PARylation with PARP inhibitors: clinically approved PARP inhibitors (olaparib, niraparib, rucaparib) serve as the foundational therapy, while the combination of PARP inhibitors with Aurora kinase A inhibitors, or concurrent PARG and CHK1 blockade, exerts synergistic anti-tumor effects by inducing lethal replication stress and metabolic collapse in cancer cells. (2) Targeting PTM-associated resistance mechanisms: this section covers emerging therapeutic strategies including disrupting the ATR/CHK1 pathway to abrogate DNA damage response and cell cycle control, targeting tumor microenvironment components via modulating JNK/p38 MAPK signaling, and triggering ferroptosis through GPX4 inhibition to eliminate drug-resistant ovarian cancer cells. (3) Combination therapies to overcome acquired resistance: core combinatorial regimens are summarized, including PARP inhibitors combined with ATR inhibitors to induce replication fork collapse and DNA double-strand breaks and trigger cancer cell apoptosis; dual targeting of metabolic and DNA repair crosstalk via small-molecule combinations (WEE1 kinase suppressors, BET domain inhibitors); and surface marker-specific depletion of cancer stem cells (CSCs) to eradicate the root of tumor recurrence and drug resistance. Collectively, this figure illustrates the multi-level therapeutic strategies targeting PARylation and PTM networks to improve clinical outcomes of ovarian cancer patients.

Small-molecule combinations exploiting synthetic lethality manifest preclinical synergy with PARP inhibitors. These include WEE1 kinase suppressors and BET domain inhibitors, which collectively amplify DNA damage to overcome adaptive resistance (214). Collectively, PARylation-centered combinatorial strategies—including DNA repair sabotage, metabolic disruption, immunomodulation and CSC eradication, and advanced drug delivery—could counter ovarian cancer’s evolvable defenses, provided toxicity profiles prove manageable in clinical translation (**Figure 2**).

Despite the promise of combination strategies, several challenges remain. The optimal sequencing and dosing of agents to maximize synergy while minimizing overlapping toxicities are not yet defined. Patient selection based on predictive biomarkers—such as HRD status, immune infiltrate profiles, or specific PTM signatures—will be critical to avoid unnecessary toxicity and improve risk-benefit ratios. Moreover, the emergence of resistance to combination regimens, driven by tumor heterogeneity or adaptive rewiring of PTM networks, warrants prospective investigation in well-designed clinical trials.

## 6 Challenges and future directions

In ovarian cancer, therapeutic development targeting post-translational modifications involves both opportunities and substantial challenges due to the intricate nature of these biochemical changes. Phosphorylation, ubiquitination, acetylation, and glycosylation are key types of post-translational modifications that introduce regulatory layers researchers might exploit for therapy. Yet the dynamic reversibility and context-dependence of these alterations create obstacles for effective treatment design. A major difficulty lies in pinpointing modifications critical for cancer progression and survival, particularly given the highly complex tumor microenvironment in ovarian malignancies. This environment features interactions between cancer cells and diverse stromal and immune cells, which shape modification patterns like phosphorylation states of signaling proteins; such states modulate pathways driving cancer advancement and therapy resistance (215). To achieve therapeutic relevance, scientists must unravel these cellular interactions. Developing selective inhibitors that target aberrant modifications while sparing normal processes poses another hurdle, as specificity is vital to minimize off-target effects and systemic toxicity. Proteolysis-targeting chimeras (PROTACs) offer a novel strategy for degrading proteins bearing specific modifications, which could counter drug resistance and boost selectivity in cancer treatment (216, 217). Although PROTACs show efficacy, optimizing their design and delivery for precise targeting in ovarian cancer requires further validation to address existing limitations.

Redundancy and compensatory mechanisms in modification pathways constrain therapy efficacy. Inhibiting one pathway may activate alternatives that restore function, diminishing therapeutic impact and demanding a thorough grasp of modification networks and their signaling cross-talk in ovarian cancer (196, 218). Combination therapies attacking multiple modification pathways or integrating agents with chemotherapy or immunotherapy could prove more effective against such issues. Moreover, reliable biomarkers to monitor modification status and predict response are critical for implementing targeted therapies, helping identify patients who benefit most from specific strategies and guiding decisions. However, discovering and validating these biomarkers in ovarian cancer remains at an early stage, necessitating additional research and clinical trials (219). While targeting post-translational modifications in ovarian cancer presents formidable obstacles, it holds considerable potential for advancing therapeutic strategies. Progress in understanding the role of modifications in cancer biology, alongside approaches like PROTACs and combination therapies, may improve patient outcomes. Continued investigation into modification mechanisms and their interplay with cellular processes will be key to overcoming challenges and unlocking full therapeutic benefits (220, 221).

Equally challenging is the management of overlapping toxicities when combining agents with distinct but intersecting mechanisms. PARP inhibitors are associated with hematologic toxicities, while ATR or CHK1 inhibitors can exacerbate myelosuppression, and immune checkpoint inhibitors carry risks of immune-related adverse events (222–227). The optimal sequencing, dosing, and schedule of combination regimens to maximize synergy while minimizing cumulative toxicity have not been established and will require careful dose-finding studies and adaptive trial designs.

Beyond tolerability, the inevitable emergence of resistance to combination therapies poses a long-term concern. Tumor cells may adapt by rewiring PTM networks, upregulating drug efflux pumps, or activating bypass signaling pathways. For example, acquired resistance to PARP inhibitor-based combinations could involve restoration of homologous recombination through secondary BRCA mutations, stabilization of replication forks, or metabolic reprogramming that mitigates replication stress (65, 228–232). Understanding these adaptive mechanisms will be critical to developing sequential or intermittent dosing strategies that forestall resistance.

Emerging approaches addressing distinct resistance mechanisms and therapeutic targets have potential in ovarian cancer treatment. Co-delivery of MDR1 and BCL2 siRNA using PLGA nanoparticles targets interdependent drug efflux and anti-apoptotic pathways in multidrug-resistant tumors. Simultaneous inhibition of these pathways enhances chemotherapy efficacy, potentially overcoming paclitaxel and cisplatin resistance in ovarian cancer cells (233). Blocking the Oncostatin M receptor (OSMR), critical for cisplatin resistance, disrupts STAT3-mediated integrin signaling to reverse chemoresistance, emphasizing targeted therapy’s role in improving outcomes (234).

Although PARP inhibitors substantially improved ovarian cancer prognosis, rising resistance necessitates new solutions. Current efforts focus on deciphering homologous recombination recovery mechanisms and developing combination therapies to bolster PARP inhibitor efficacy, aiming to delay recurrence and enhance clinical benefits (235). In HGSOC, targeting estrogen metabolism offers an alternative strategy against platinum resistance. Identifying estrogen biosynthesis-related genes as prognostic biomarkers enables therapies that restrict estrogen-driven proliferation, addressing HGSOC treatment unresponsiveness (236). Downregulating hypoxia-inducible factor-1 (HIF-1) constitutes another avenue for countering cisplatin resistance. Suppressing HIF-1 redirects cancer cell metabolism from glycolysis toward mitochondrial oxidative phosphorylation, increasing reactive oxygen species production that induces cell death. This metabolic shift proves lethal to resistant cells *in vitro* (237). Endoplasmic reticulum stress induction through unfolded protein response (UPR) activation also has therapeutic potential. Triggering UPR-mediated apoptosis in resistant ovarian cancer cells provides a mechanistic basis for overcoming multidrug resistance (238). These advances, while requiring further validation, may ultimately enhance treatment efficacy for ovarian cancer patients facing therapeutic challenges.

Advancing therapeutic outcomes in ovarian cancer requires prioritizing fundamental research on resistance mechanisms, with molecular pathway elucidation serving as the cornerstone. Deciphering drug resistance drivers enables targeted intervention development; for instance, PI3K/AKT/mTOR pathway activation contributes substantially to chemotherapy unresponsiveness, positioning pathway inhibitors as viable therapeutic candidates (171). Parallel investigation of JNK/p38 MAPK signaling displays its chemoresistance involvement, suggesting pathway blockade could restore treatment sensitivity (197). Beyond cell-autonomous mechanisms, the tumor microenvironment constitutes an important research frontier. Cancer-associated fibroblast interactions with malignant cells promote tumor progression and resistance via PI3K-Akt and other signaling cascades (239). Disrupting these stromal collaborations may undermine microenvironment-mediated protection.

Epigenetic and post-transcriptional regulators offer additional investigative dimensions. MicroRNA dysregulation influences chemoresistance through pathway modulation, establishing miRNA targeting as a potential therapeutic strategy (195). Similarly, epigenetic alterations like DNA methylation and histone modifications drive resistance phenotypes, warranting exploration as both biomarkers and intervention targets (240). Technological innovation represents another priority, where nanoparticle-based delivery systems may overcome resistance by precisely targeting mechanistic vulnerabilities (198). Liquid biopsy biomarkers concurrently enable non-invasive monitoring of therapeutic response and resistance evolution, facilitating personalized management (241).

Cancer stem cell biology demands particular attention given its role in metastasis and treatment failure. Understanding stem cell-driven recurrence mechanisms could yield therapies preventing relapse (242). Looking forward, the integration of multi-omics profiling—including genomics, transcriptomics, proteomics, and PTM-specific analyses—will be essential to map the dynamic networks that underpin resistance. Spatiotemporal resolution of PTM changes during tumor progression and treatment will require advanced imaging technologies and longitudinal sampling of patient tumors. Collaborative efforts to establish standardized protocols for PTM biomarker validation and to design rationally sequenced combination trials will be critical to translating mechanistic insights into durable clinical benefits. These research vectors including molecular mechanisms, microenvironment crosstalk, regulatory networks, technological solutions and stem cell biology must integrate to develop effective countermeasures against ovarian cancer resistance. While substantial challenges persist, coordinated investigation across these domains offers a viable path toward improved survival.

## 7 Conclusions

This synthesis establishes PARylation as a central orchestrator in ovarian cancer pathogenesis, governing DNA repair fidelity, immune microenvironment remodeling, and therapeutic resistance through dynamic crosstalk with phosphorylation, ubiquitination, and epigenetic modifications. PARP1/2-mediated signaling suppresses cGAS/STING-dependent antitumor immunity while enabling chemoresistance via PTM networks involving EHMT1/2-driven histone methylation, SPINDOC-enhanced PARylation, and ubiquitin-dependent stabilization pathways. Critically, the convergence of PARylation with SUMOylation and metabolic stress responses creates adaptive resilience mechanisms that compromise PARP inhibitor efficacy. Overcoming this multidimensional resistance requires integrated strategies targeting PARylation-immune crosstalk, epigenetic reprogramming, and PTM-regulated DNA damage metabolism. Nanoparticle delivery systems and PROTACs represent rational approaches for disrupting “undruggable” nodes within these networks, though clinical translation necessitates resolving spatiotemporal PTM dynamics in tumor-stroma interactions and validating liquid biopsy biomarkers. Concerted efforts to elucidate cancer stem cell-specific PTM dependencies and combinatorial targeting paradigms offer a viable path toward countering ovarian cancer’s evolvable defenses.

## References

[1] Chen L Liu S Tao Y . Regulating tumor suppressor genes: post-translational modifications. Sig Transduct Target Ther. (2020) 5:90. doi: 10.1038/s41392-020-0196-9. PMID: 32532965 PMC7293209

[2] Mowen KA David M . Unconventional post-translational modifications in immunological signaling. Nat Immunol. (2014) 15:512–20. doi: 10.1038/ni.2873. PMID: 24840982

[3] Miao C Huang Y Zhang C Wang X Wang B Zhou X . Post-translational modifications in drug resistance. Drug Resistance Updates. (2025) 78:101173. doi: 10.1016/j.drup.2024.101173. PMID: 39612546

[4] Hitosugi T Chen J . Post-translational modifications and the Warburg effect. Oncogene. (2014) 33:4279–85. doi: 10.1038/onc.2013.406. PMID: 24096483

[5] Lin Y Lin P Lu Y Zheng J Zheng Y Huang X . Post‐translational modifications of RNA‐modifying proteins in cellular dynamics and disease progression. Adv Sci. (2024) 11:e2406318. doi: 10.1002/advs.202406318. PMID: 39377984 PMC11600222

[6] Blomen VA Boonstra J . Stable transmission of reversible modifications: maintenance of epigenetic information through the cell cycle. Cell Mol Life Sci. (2011) 68:27–44. doi: 10.1007/s00018-010-0505-5. PMID: 20799050 PMC3015210

[7] Wu Z Huang R Yuan L . Crosstalk of intracellular post-translational modifications in cancer. Arch Biochem Biophys. (2019) 676:108138. doi: 10.1016/j.abb.2019.108138. PMID: 31606391

[8] Schwarz SD Xu J Gunasekera K Schürmann D Vågbø CB Ferrari E . Covalent PARylation of DNA base excision repair proteins regulates DNA demethylation. Nat Commun. (2024) 15:184. doi: 10.1038/s41467-023-44209-8. PMID: 38167803 PMC10762122

[9] Yang J Wan S Song Q Xie Y Wan J Zhou Y . Angiopoietin-like protein 8 directs DNA damage responses towards apoptosis by stabilizing PARP1-DNA condensates. Cell Death Differ. (2025) 32:672–88. doi: 10.1038/s41418-024-01422-2. PMID: 39592710 PMC11982567

[10] Gupte R Liu Z Kraus WL . PARPs and ADP-ribosylation: recent advances linking molecular functions to biological outcomes. Genes Dev. (2017) 31:101–26. doi: 10.1101/gad.291518.116. PMID: 28202539 PMC5322727

[11] Zhang Y Xiong X Zhu Q Zhang J Chen S Wang Y . FER-mediated phosphorylation and PIK3R2 recruitment on IRS4 promotes AKT activation and tumorigenesis in ovarian cancer cells. Elife. (2022) 11:e76183. doi: 10.7554/eLife.76183. PMID: 35550247 PMC9098222

[12] Chen J Shan W Jia Q Chen Y Jiang W Tian Y . USP33 facilitates the ovarian cancer progression via deubiquitinating and stabilizing CBX2. Oncogene. (2024) 43:3170–83. doi: 10.1038/s41388-024-03151-9. PMID: 39256572

[13] Xia L Mei J Huang M Bao D Wang Z Chen Y . O-GlcNAcylation in ovarian tumorigenesis and its therapeutic implications. Transl Oncol. (2025) 51:102220. doi: 10.1016/j.tranon.2024.102220. PMID: 39616984 PMC11647086

[14] Marsh DJ Shah JS Cole AJ . Histones and their modifications in ovarian cancer-drivers of disease and therapeutic targets. Front Oncol. (2014) 4:144. doi: 10.3389/fonc.2014.00144. PMID: 24971229 PMC4053763

[15] Martincuks A Song J Kohut A Zhang C Li Y-J Zhao Q . PARP inhibition activates STAT3 in both tumor and immune cells underlying therapy resistance and immunosuppression in ovarian cancer. Front Oncol. (2021) 11:724104. doi: 10.3389/fonc.2021.724104. PMID: 34956861 PMC8693573

[16] Zuo W-W Zhao C-F Li Y Sun H-Y Ma G-M Liu Y-P . High expression of PARP1 in tumor and stroma cells predicts different prognosis and platinum resistance in patients with advanced epithelial ovarian cancer. Front Oncol. (2022) 12:931445. doi: 10.3389/fonc.2022.931445. PMID: 35875162 PMC9301997

[17] Turinetto M Scotto G Tuninetti V Giannone G Valabrega G . The role of PARP inhibitors in the ovarian cancer microenvironment: moving forward from synthetic lethality. Front Oncol. (2021) 11:689829. doi: 10.3389/fonc.2021.689829. PMID: 34195090 PMC8238121

[18] Hooda J Novak M Salomon MP Matsuba C Ramos RI MacDuffie E . Early loss of histone H2B monoubiquitylation alters chromatin accessibility and activates key immune pathways that facilitate progression of ovarian cancer. Cancer Res. (2019) 79:760–72. doi: 10.1158/0008-5472.CAN-18-2297. PMID: 30563893 PMC6377833

[19] Song G Chen L Zhang B Song Q Yu Y Moore C . Proteome-wide tyrosine phosphorylation analysis reveals dysregulated signaling pathways in ovarian tumors. Mol Cell Proteomics. (2019) 18:448–60. doi: 10.1074/mcp.RA118.000851. PMID: 30523211 PMC6398206

[20] Liu Y Liu H Ye M Jiang M Chen X Song G . Methylation of BRD4 by PRMT1 regulates BRD4 phosphorylation and promotes ovarian cancer invasion. Cell Death Dis. (2023) 14:624. doi: 10.1038/s41419-023-06149-5. PMID: 37737256 PMC10517134

[21] Shimizu K Gi M Suzuki S North BJ Watahiki A Fukumoto S . Interplay between protein acetylation and ubiquitination controls MCL1 protein stability. Cell Rep. (2021) 37:109988. doi: 10.1016/j.celrep.2021.109988. PMID: 34758305 PMC8621139

[22] Saldova R Dempsey E Pérez-Garay M Mariño K Watson JA Blanco-Fernández A . 5-AZA-2’-deoxycytidine induced demethylation influences N-glycosylation of secreted glycoproteins in ovarian cancer. Epigenetics. (2011) 6:1362–72. doi: 10.4161/epi.6.11.17977. PMID: 22086115

[23] Mirza MR Monk BJ Herrstedt J Oza AM Mahner S Redondo A . Niraparib maintenance therapy in platinum-sensitive, recurrent ovarian cancer. N Engl J Med. (2016) 375:2154–64. doi: 10.1056/NEJMoa1611310. PMID: 27717299

[24] Moore K Colombo N Scambia G Kim B-G Oaknin A Friedlander M . Maintenance olaparib in patients with newly diagnosed advanced ovarian cancer. N Engl J Med. (2018) 379:2495–505. doi: 10.1056/NEJMoa1810858. PMID: 30345884

[25] Patch A-M Christie EL Etemadmoghadam D Garsed DW George J Fereday S . Whole–genome characterization of chemoresistant ovarian cancer. Nature. (2015) 521:489–94. doi: 10.1038/nature14410. PMID: 26017449

[26] Beneyton A Nonfoux L Gagné J-P Rodrigue A Kothari C Atalay N . The dynamic process of covalent and non-covalent PARylation in the maintenance of genome integrity: a focus on PARP inhibitors. NAR Cancer. (2023) 5:zcad043. doi: 10.1093/narcan/zcad043. PMID: 37609662 PMC10440794

[27] Gogola E Duarte AA de Ruiter JR Wiegant WW Schmid JA de Bruijn R . Selective loss of PARG restores PARylation and counteracts PARP inhibitor-mediated synthetic lethality. Cancer Cell. (2018) 33:1078–1093.e12. doi: 10.1016/j.ccell.2018.05.008. PMID: 29894693

[28] Nespolo A Stefenatti L Pellarin I Gambelli A Rampioni Vinciguerra GL Karimbayli J . USP1 deubiquitinates PARP1 to regulate its trapping and PARylation activity. Sci Adv. (2024) 10:eadp6567. doi: 10.1126/sciadv.adp6567. PMID: 39536107 PMC11559621

[29] Miao H Meng H Zhang Y Chen T Zhang L Cheng W . FSP1 inhibition enhances olaparib sensitivity in BRCA-proficient ovarian cancer patients via a nonferroptosis mechanism. Cell Death Differ. (2024) 31:497–510. doi: 10.1038/s41418-024-01263-z. PMID: 38374229 PMC11043371

[30] Xia Y Huang P Qian Y Wang Z Jin N Li X . PARP inhibitors enhance antitumor immune responses by triggering pyroptosis via TNF–caspase 8–GSDMD/E axis in ovarian cancer. J Immunother Cancer. (2024) 12:e009032. doi: 10.1136/jitc-2024-009032. PMID: 39366751 PMC11459312

[31] Nguyen LL Watson ZL Ortega R Woodruff ER Jordan KR Iwanaga R . EHMT1/2 inhibition promotes regression of therapy-resistant ovarian cancer tumors in a CD8 T-cell–dependent manner. Mol Cancer Res. (2024) 22:1117–27. doi: 10.1158/1541-7786.MCR-24-0067. PMID: 39136655 PMC11614706

[32] Jeanne A Sarazin T Charlé M Moali C Fichel C Boulagnon-Rombi C . Targeting ovarian carcinoma with TSP-1:CD47 antagonist TAX2 activates anti-tumor immunity. Cancers. (2021) 13:5019. doi: 10.3390/cancers13195019. PMID: 34638503 PMC8508526

[33] Wang L Wang D Sonzogni O Ke S Wang Q Thavamani A . PARP-inhibition reprograms macrophages toward an anti-tumor phenotype. Cell Rep. (2022) 41:111462. doi: 10.1016/j.celrep.2022.111462. PMID: 36223740 PMC9727835

[34] Shu Y Jin X Ji M Zhang Z Wang X Liang H . Ku70 binding to YAP alters PARP1 ubiquitination to regulate genome stability and tumorigenesis. Cancer Res. (2024) 84:2836–55. doi: 10.1158/0008-5472.CAN-23-4034. PMID: 38862269

[35] Zhang N Zhang Y Qian H Wu S Cao L Sun Y . Selective targeting of ubiquitination and degradation of PARP1 by E3 ubiquitin ligase WWP2 regulates isoproterenol-induced cardiac remodeling. Cell Death Differ. (2020) 27:2605–19. doi: 10.1038/s41418-020-0523-2. PMID: 32139900 PMC7429876

[36] Zhang F-L Yang S-Y Liao L Zhang T-M Zhang Y-L Hu S-Y . Dynamic SUMOylation of MORC2 orchestrates chromatin remodelling and DNA repair in response to DNA damage and drives chemoresistance in breast cancer. Theranostics. (2023) 13:973–90. doi: 10.7150/thno.79688. PMID: 36793866 PMC9925317

[37] Borgermann N Ackermann L Schwertman P Hendriks IA Thijssen K Liu JC . SUMOylation promotes protective responses to DNA-protein crosslinks. EMBO J. (2019) 38:e101496. doi: 10.15252/embj.2019101496. PMID: 30914427 PMC6463212

[38] Liu J Zhang S Cao L Zhang N Guo Q Zou Y . The deubiquitination-PARylation positive feedback loop of the USP10-PARP1 axis promotes DNA damage repair and affects therapeutic efficacy of PARP1 inhibitor. Oncogene. (2025) 44:2515–29. doi: 10.1038/s41388-025-03428-7. PMID: 40316740 PMC12256264

[39] Geenen JJJ Schellens JHM . Molecular pathways: targeting the protein kinase Wee1 in cancer. Clin Cancer Res. (2017) 23:4540–4. doi: 10.1158/1078-0432.CCR-17-0520. PMID: 28442503

[40] Teo ZL O’Connor MJ Versaci S Clarke KA Brown ER Percy LW . Combined PARP and WEE1 inhibition triggers anti-tumor immune response in BRCA1/2 wildtype triple-negative breast cancer. NPJ Breast Cancer. (2023) 9:68. doi: 10.1038/s41523-023-00568-5. PMID: 37582853 PMC10427618

[41] Yang B Li X Fu Y Guo E Ye Y Li F . MEK inhibition remodels the immune landscape of mutant KRAS tumors to overcome resistance to PARP and immune checkpoint inhibitors. Cancer Res. (2021) 81:2714–29. doi: 10.1158/0008-5472.CAN-20-2370. PMID: 33589518 PMC8265237

[42] Sun C Fang Y Yin J Chen J Ju Z Zhang D . Rational combination therapy with PARP and MEK inhibitors capitalizes on therapeutic liabilities in RAS mutant cancers. Sci Transl Med. (2017) 9:eaal5148. doi: 10.1126/scitranslmed.aal5148. PMID: 28566428 PMC5919217

[43] Awasthi S Dobrolecki LE Sallas C Zhang X Li Y Khazaei S . UBA1 inhibition sensitizes cancer cells to PARP inhibitors. Cell Rep Med. (2024) 5:101834. doi: 10.1016/j.xcrm.2024.101834. PMID: 39626673 PMC11722100

[44] Barghout SH Patel PS Wang X Xu GW Kavanagh S Halgas O . Preclinical evaluation of the selective small-molecule UBA1 inhibitor, TAK-243, in acute myeloid leukemia. Leukemia. (2019) 33:37–51. doi: 10.1038/s41375-018-0167-0. PMID: 29884901

[45] Zou Y Zhang H Chen P Tang J Yang S Nicot C . Clinical approaches to overcome PARP inhibitor resistance. Mol Cancer. (2025) 24:156. doi: 10.1186/s12943-025-02355-1. PMID: 40442774 PMC12123805

[46] Li Y Dobrolecki LE Sallas C Zhang X Kerr TD Bisht D . PRMT blockade induces defective DNA replication stress response and synergizes with PARP inhibition. Cell Rep Med. (2023) 4:101326. doi: 10.1016/j.xcrm.2023.101326. PMID: 38118413 PMC10772459

[47] Aparnathi MK Ul Haq S St-Germain J Nixon KCJ Walton J Song L . PRMT1 inhibitor MS023 suppresses RNA splicing to sensitize small cell lung cancer to DNA damaging agents. Neoplasia. (2025) 66:101176. doi: 10.1016/j.neo.2025.101176. PMID: 40413955 PMC12152348

[48] Robert I Karicheva O Reina San Martin B Schreiber V Dantzer F . Functional aspects of PARylation in induced and programmed DNA repair processes: preserving genome integrity and modulating physiological events. Mol Aspects Med. (2013) 34:1138–52. doi: 10.1016/j.mam.2013.02.001. PMID: 23454615

[49] Alemasova EE Lavrik OI . Poly(ADP-ribosyl)ation by PARP1: reaction mechanism and regulatory proteins. Nucleic Acids Res. (2019) 47:3811–27. doi: 10.1093/nar/gkz120. PMID: 30799503 PMC6486540

[50] Konstantinopoulos PA Spentzos D Karlan BY Taniguchi T Fountzilas E Francoeur N . Gene expression profile of BRCAness that correlates with responsiveness to chemotherapy and with outcome in patients with epithelial ovarian cancer. Jco. (2010) 28:3555–61. doi: 10.1200/JCO.2009.27.5719. PMID: 20547991 PMC2917311

[51] Kristeleit R Lisyanskaya A Fedenko A Dvorkin M de Melo AC Shparyk Y . Rucaparib versus standard-of-care chemotherapy in patients with relapsed ovarian cancer and a deleterious BRCA1 or BRCA2 mutation (ARIEL4): an international, open-label, randomised, phase 3 trial. Lancet Oncol. (2022) 23:465–78. doi: 10.1016/S1470-2045(22)00122-X. PMID: 35298906

[52] Ledermann J Harter P Gourley C Friedlander M Vergote I Rustin G . Olaparib maintenance therapy in patients with platinum-sensitive relapsed serous ovarian cancer: a preplanned retrospective analysis of outcomes by BRCA status in a randomised phase 2 trial. Lancet Oncol. (2014) 15:852–61. doi: 10.1016/S1470-2045(14)70228-1. PMID: 24882434

[53] Oza AM Lisyanskaya A Fedenko A de Melo AC Shparyk Y Rakhmatullina I . Rucaparib versus chemotherapy for treatment of relapsed ovarian cancer with deleterious BRCA1 or BRCA2 mutation (ARIEL4): final results of an international, open-label, randomised, phase 3 trial. Lancet Oncol. (2025) 26:249–64. doi: 10.1016/S1470-2045(24)00674-0. PMID: 39914419

[54] DiSilvestro P Banerjee S Colombo N Scambia G Kim B-G Oaknin A . Overall survival with maintenance olaparib at a 7-year follow-up in patients with newly diagnosed advanced ovarian cancer and a BRCA mutation: the SOLO1/GOG 3004 trial. Jco. (2023) 41:609–17. doi: 10.1200/JCO.22.01549. PMID: 36082969 PMC9870219

[55] Duan Y Du A Gu J Duan G Wang C Gui X . PARylation regulates stress granule dynamics, phase separation, and neurotoxicity of disease-related RNA-binding proteins. Cell Res. (2019) 29:233–47. doi: 10.1038/s41422-019-0141-z. PMID: 30728452 PMC6460439

[56] Wu S Li X Gao F de Groot JF Koul D Yung WKA . PARP-mediated PARylation of MGMT is critical to promote repair of temozolomide-induced O6-methylguanine DNA damage in glioblastoma. Neuro-Oncology. (2021) 23:920–31. doi: 10.1093/neuonc/noab003. PMID: 33433610 PMC8168825

[57] Fisher AEO Hochegger H Takeda S Caldecott KW . Poly(ADP-ribose) polymerase 1 accelerates single-strand break repair in concert with poly(ADP-ribose) glycohydrolase. Mol Cell Biol. (2007) 27:5597–605. doi: 10.1128/MCB.02248-06. PMID: 17548475 PMC1952076

[58] Satoh MS Lindahl T . Role of poly(ADP-ribose) formation in DNA repair. Nature. (1992) 356:356–8. doi: 10.1038/356356a0. PMID: 1549180

[59] Strom CE Johansson F Uhlen M Szigyarto C-K Erixon K Helleday T . Poly (ADP-ribose) polymerase (PARP) is not involved in base excision repair but PARP inhibition traps a single-strand intermediate. Nucleic Acids Res. (2011) 39:3166–75. doi: 10.1093/nar/gkq1241. PMID: 21183466 PMC3082910

[60] Wang Y Kim NS Haince J-F Kang HC David KK Andrabi SA . Poly(ADP-ribose) (PAR) binding to apoptosis-inducing factor is critical for PAR polymerase-1–dependent cell death (parthanatos). Sci Signal. (2011) 4:ra20. doi: 10.1126/scisignal.2000902. PMID: 21467298 PMC3086524

[61] Barkauskaite E Brassington A Tan ES Warwicker J Dunstan MS Banos B . Visualization of poly(ADP-ribose) bound to PARG reveals inherent balance between exo- and endo-glycohydrolase activities. Nat Commun. (2013) 4:2164. doi: 10.1038/ncomms3164. PMID: 23917065 PMC3741636

[62] David KK . Parthanatos, a messenger of death. Front Biosci. (2009) 1116. doi: 10.2741/3297. PMID: 19273119 PMC4450718

[63] Wang Y Dawson VL Dawson TM . Poly(ADP-ribose) signals to mitochondrial AIF: a key event in parthanatos. Exp Neurol. (2009) 218:193–202. doi: 10.1016/j.expneurol.2009.03.020. PMID: 19332058 PMC2752872

[64] Pettitt SJ Krastev DB Brandsma I Dréan A Song F Aleksandrov R . Genome-wide and high-density CRISPR-Cas9 screens identify point mutations in PARP1 causing PARP inhibitor resistance. Nat Commun. (2018) 9:1849. doi: 10.1038/s41467-018-03917-2. PMID: 29748565 PMC5945626

[65] Ray Chaudhuri A Callen E Ding X Gogola E Duarte AA Lee J-E . Replication fork stability confers chemoresistance in BRCA-deficient cells. Nature. (2016) 535:382–7. doi: 10.1038/nature18325. PMID: 27443740 PMC4959813

[66] Lord CJ Ashworth A . Mechanisms of resistance to therapies targeting BRCA-mutant cancers. Nat Med. (2013) 19:1381–8. doi: 10.1038/nm.3369. PMID: 24202391

[67] Rondinelli B Gogola E Yücel H Duarte AA van de Ven M van der Sluijs R . EZH2 promotes degradation of stalled replication forks by recruiting MUS81 through histone H3 trimethylation. Nat Cell Biol. (2017) 19:1371–8. doi: 10.1038/ncb3626. PMID: 29035360

[68] Chu Y-Y Yam C Yamaguchi H Hung M-C . Biomarkers beyond BRCA: promising combinatorial treatment strategies in overcoming resistance to PARP inhibitors. J BioMed Sci. (2022) 29:86. doi: 10.1186/s12929-022-00870-7. PMID: 36284291 PMC9594904

[69] Biegała Ł Statkiewicz M Gajek A Szymczak-Pajor I Rusetska N Śliwińska A . Molecular mechanisms restoring olaparib efficacy through ATR/CHK1 pathway inhibition in olaparib-resistant BRCA1/2MUT ovarian cancer models. Biochim Biophys Acta (BBA) - Mol Basis Dis. (2025) 1871:167574. doi: 10.1016/j.bbadis.2024.167574. PMID: 39557132

[70] Gibson BA Kraus WL . New insights into the molecular and cellular functions of poly(ADP-ribose) and PARPs. Nat Rev Mol Cell Biol. (2012) 13:411–24. doi: 10.1038/nrm3376. PMID: 22713970

[71] Hassa PO Haenni SS Elser M Hottiger MO . Nuclear ADP-ribosylation reactions in mammalian cells: where are we today and where are we going? Microbiol Mol Biol Rev. (2006) 70:789–829. doi: 10.1128/MMBR.00040-05. PMID: 16959969 PMC1594587

[72] Schreiber V Dantzer F Ame J-C de Murcia G . Poly(ADP-ribose): novel functions for an old molecule. Nat Rev Mol Cell Biol. (2006) 7:517–28. doi: 10.1038/nrm1963. PMID: 16829982

[73] Krishnakumar R Kraus WL . The PARP side of the nucleus: molecular actions, physiological outcomes, and clinical targets. Mol Cell. (2010) 39:8–24. doi: 10.1016/j.molcel.2010.06.017. PMID: 20603072 PMC2923840

[74] Luo X Kraus WL . On PAR with PARP: cellular stress signaling through poly(ADP-ribose) and PARP-1. Genes Dev. (2012) 26:417–32. doi: 10.1101/gad.183509.111. PMID: 22391446 PMC3305980

[75] Hassa PO Hottiger MO . The functional role of poly(ADP-ribose)polymerase 1 as novel coactivator of NF-κB in inflammatory disorders. CMLS Cell Mol Life Sci. (2002) 59:1534–53. doi: 10.1007/s00018-002-8527-2. PMID: 12440774 PMC11337477

[76] Ji Y Tulin AV . The roles of PARP1 in gene control and cell differentiation. Curr Opin Genet Dev. (2010) 20:512–8. doi: 10.1016/j.gde.2010.06.001. PMID: 20591646 PMC2942995

[77] Kraus WL . Transcriptional control by PARP-1: chromatin modulation, enhancer-binding, coregulation, and insulation. Curr Opin Cell Biol. (2008) 20:294–302. doi: 10.1016/j.ceb.2008.03.006. PMID: 18450439 PMC2518631

[78] Kim MY Zhang T Kraus WL . Poly(ADP-ribosyl)ation by PARP-1: ‘PAR-laying’ NAD^+^ into a nuclear signal. Genes Dev. (2005) 19:1951–67. doi: 10.1101/gad.1331805. PMID: 16140981

[79] Rouleau M Patel A Hendzel MJ Kaufmann SH Poirier GG . PARP inhibition: PARP1 and beyond. Nat Rev Cancer. (2010) 10:293–301. doi: 10.1038/nrc2812. PMID: 20200537 PMC2910902

[80] Cohen-Armon M . PARP-1 activation in the ERK signaling pathway. Trends Pharmacol Sci. (2007) 28:556–60. doi: 10.1016/j.tips.2007.08.005. PMID: 17950909

[81] Underhill C Toulmonde M Bonnefoi H . A review of PARP inhibitors: from bench to bedside. Ann Oncol. (2011) 22:268–79. doi: 10.1093/annonc/mdq322. PMID: 20643861

[82] Schreiber V Amé J-C Dollé P Schultz I Rinaldi B Fraulob V . Poly(ADP-ribose) polymerase-2 (PARP-2) is required for efficient base excision DNA repair in association with PARP-1 and XRCC1. J Biol Chem. (2002) 277:23028–36. doi: 10.1074/jbc.M202390200. PMID: 11948190

[83] Ray Chaudhuri A Nussenzweig A . The multifaceted roles of PARP1 in DNA repair and chromatin remodelling. Nat Rev Mol Cell Biol. (2017) 18:610–21. doi: 10.1038/nrm.2017.53. PMID: 28676700 PMC6591728

[84] Obaji E Haikarainen T Lehtiö L . Structural basis for DNA break recognition by ARTD2/PARP2. Nucleic Acids Res. (2018) 46:12154–65. doi: 10.1093/nar/gky927. PMID: 30321391 PMC6294510

[85] Chen Q Kassab MA Dantzer F Yu X . PARP2 mediates branched poly ADP-ribosylation in response to DNA damage. Nat Commun. (2018) 9:3233. doi: 10.1038/s41467-018-05588-5. PMID: 30104678 PMC6089979

[86] Krastev DB Pettitt SJ Campbell J Song F Tanos BE Stoynov SS . Coupling bimolecular PARylation biosensors with genetic screens to identify PARylation targets. Nat Commun. (2018) 9:2016. doi: 10.1038/s41467-018-04466-4. PMID: 29789535 PMC5964205

[87] Chang P Coughlin M Mitchison TJ . Tankyrase-1 polymerization of poly(ADP-ribose) is required for spindle structure and function. Nat Cell Biol. (2005) 7:1133–9. doi: 10.1038/ncb1322. PMID: 16244666

[88] Smith S Giriat I Schmitt A de Lange T . Tankyrase, a poly(ADP-ribose) polymerase at human telomeres. Science. (1998) 282:1484–7. doi: 10.1126/science.282.5393.1484. PMID: 9822378

[89] Yang L Sun L Teng Y Chen H Gao Y Levine AS . Tankyrase1-mediated poly(ADP-ribosyl)ation of TRF1 maintains cell survival after telomeric DNA damage. Nucleic Acids Res. (2017) 45:3906–21. doi: 10.1093/nar/gkx083. PMID: 28160604 PMC5397190

[90] Li N Wang Y Neri S Zhen Y Fong LWR Qiao Y . Tankyrase disrupts metabolic homeostasis and promotes tumorigenesis by inhibiting LKB1-AMPK signalling. Nat Commun. (2019) 10:4363. doi: 10.1038/s41467-019-12377-1. PMID: 31554794 PMC6761205

[91] Kraus WL . PARPs and ADP-ribosylation: 50 years … and counting. Mol Cell. (2015) 58:902–10. doi: 10.1016/j.molcel.2015.06.006. PMID: 26091339 PMC4477203

[92] Weixler L Ikenga NJ Voorneveld J Aydin G Bolte TM Momoh J . Protein and RNA ADP-ribosylation detection is influenced by sample preparation and reagents used. Life Sci Alliance. (2023) 6:e202201455. doi: 10.26508/lsa.202201455. PMID: 36368907 PMC9652768

[93] Gao F Zhao R Huang L Yi X . Background-quenched aggregation-induced emission through electrostatic interactions for the detection of poly(ADP-ribose) polymerase-1 activity. Molecules. (2023) 28:4759. doi: 10.3390/molecules28124759. PMID: 37375313 PMC10302931

[94] Furman JL Mok P-W Shen S Stains CI Ghosh I . A turn-on split-luciferase sensor for the direct detection of poly(ADP-ribose) as a marker for DNA repair and cell death. Chem Commun. (2011) 47:397–9. doi: 10.1039/C0CC02229B. PMID: 20830433

[95] Gao F Liu G Qiao Y Dong X Liu L . Streptavidin-conjugated DNA for the boronate affinity-based detection of poly(ADP-ribose) polymerase-1 with improved sensitivity. Biosensors. (2023) 13:723. doi: 10.3390/bios13070723. PMID: 37504121 PMC10377026

[96] Liu Y Fan J Shangguan L Liu Y Wei Y Wei W . Ultrasensitive electrochemical detection of poly (ADP-ribose) polymerase-1 via polyaniline deposition. Talanta. (2018) 180:127–32. doi: 10.1016/j.talanta.2017.11.072. PMID: 29332790

[97] Koczor CA Haider AJ Saville KM Li J Andrews JF Beiser AV . Live cell detection of poly(ADP-ribose) for use in genetic and genotoxic compound screens. Cancers. (2022) 14:3676. doi: 10.3390/cancers14153676. PMID: 35954352 PMC9367489

[98] Liu Y Fan J Yang H Xu E Wei W Zhang Y . Detection of PARP-1 activity based on hyperbranched-poly (ADP-ribose) polymers responsive current in artificial nanochannels. Biosens Bioelectron. (2018) 113:136–41. doi: 10.1016/j.bios.2018.05.005. PMID: 29754052

[99] Yang S Huang Y Yang T Li J Tian J Liu L . Electrochemical detection of poly(ADP-ribose) polymerase-1 with silver nanoparticles as signal labels by integrating the advantages of homogeneous reaction with surface-tethered detection. Talanta. (2025) 281:126796. doi: 10.1016/j.talanta.2024.126796. PMID: 39226698

[100] Chiu S-P Camacho CV Kraus WL . Development and characterization of recombinant ADP-ribose binding reagents that allow simultaneous detection of mono and poly ADP-ribose. J Biol Chem. (2024) 300:107609. doi: 10.1016/j.jbc.2024.107609. PMID: 39074634 PMC11388009

[101] Wang C Liu A Chen J Liu S Wei W . Sensitive detection of PARP-1 activity by electrochemical impedance spectroscopy based on biomineralization. Anal Chim Acta. (2023) 1249:340937. doi: 10.1016/j.aca.2023.340937. PMID: 36868772

[102] Dasovich M Leung AKL . PARPs and ADP-ribosylation: Deciphering the complexity with molecular tools. Mol Cell. (2023) 83:1552–72. doi: 10.1016/j.molcel.2023.04.009. PMID: 37119811 PMC10202152

[103] Challa S Ryu KW Whitaker AL Abshier JC Camacho CV Kraus WL . Development and characterization of new tools for detecting poly(ADP-ribose) *in vitro* and *in vivo*. Elife. (2022) 11:e72464. doi: 10.7554/eLife.72464. PMID: 35476036 PMC9045816

[104] Puentes LN Makvandi M Mach RH . Molecular imaging: PARP-1 and beyond. J Nucl Med. (2021) 62:765–70. doi: 10.2967/jnumed.120.243287. PMID: 33579802

[105] Pandey N Black BE . Rapid detection and signaling of DNA damage by PARP-1. Trends Biochem Sci. (2021) 46:744–57. doi: 10.1016/j.tibs.2021.01.014. PMID: 33674152 PMC8364484

[106] Zhou X Wang C Wang Z Yang H Wei W Liu Y . Renewable electrochemical sensor for PARP-1 activity detection based on host-guest recognition. Biosens Bioelectron. (2020) 148:111810. doi: 10.1016/j.bios.2019.111810. PMID: 31710960

[107] Zhang D Wang K Wei W Liu S . Single-particle assay of poly(ADP-ribose) polymerase-1 activity with dark-field optical microscopy. ACS Sens. (2020) 5:1198–206. doi: 10.1021/acssensors.0c00264. PMID: 32208631

[108] Yang H Fu F Li W Wei W Zhang Y Liu S . Telomerase and poly(ADP-ribose) polymerase-1 activity sensing based on the high fluorescence selectivity and sensitivity of TOTO-1 towards G bases in single-stranded DNA and poly(ADP-ribose). Chem Sci. (2019) 10:3706–14. doi: 10.1039/C8SC05770B. PMID: 31015914 PMC6461019

[109] Wang Z Xu E Wang C Wei W Liu Y Liu S . High specificity and efficiency electrochemical detection of poly(ADP-ribose) polymerase-1 activity based on versatile peptide-templated copper nanoparticles and detection array. Anal Chim Acta. (2019) 1091:95–102. doi: 10.1016/j.aca.2019.09.023. PMID: 31679579

[110] Wang C Li Y Xu E Zhou Q Chen J Wei W . A label-free PFP-based photoelectrochemical biosensor for highly sensitive detection of PARP-1 activity. Biosens Bioelectron. (2019) 138:111308. doi: 10.1016/j.bios.2019.05.013. PMID: 31103013

[111] Liu Y Xu X Yang H Xu E Wu S Wei W . Analysis of poly(ADP-ribose) polymerase-1 by enzyme-initiated auto-PARylation-controlled aggregation of hemin-graphene nanocomposites. Analyst. (2018) 143:2501–7. doi: 10.1039/C8AN00009C. PMID: 29664094

[112] Bilan V Selevsek N Kistemaker HAV Abplanalp J Feurer R Filippov DV . New quantitative mass spectrometry approaches reveal different ADP-ribosylation phases dependent on the levels of oxidative stress. Mol Cell Proteomics. (2017) 16:949–58. doi: 10.1074/mcp.O116.065623. PMID: 28325851 PMC5417832

[113] Xu Y Liu L Wang Z Dai Z . Stable and reusable electrochemical biosensor for poly(ADP-ribose) polymerase and its inhibitor based on enzyme-initiated auto-PARylation. ACS Appl Mater Interfaces. (2016) 8:18669–74. doi: 10.1021/acsami.6b01883. PMID: 27367274

[114] Thomas A Upadhyaya K Bejan D Adoff H Cohen M Schultz C . A genetically encoded sensor for real-time monitoring of poly-ADP-ribosylation dynamics *in vitro* and in cells. ACS Sens. (2024) 9:5246–52. doi: 10.1021/acssensors.4c01406. PMID: 39351594 PMC11520908

[115] Serebrovskaya EO Podvalnaya NM Dudenkova VV Efremova AS Gurskaya NG Gorbachev DA . Genetically encoded fluorescent sensor for poly-ADP-ribose. IJMS. (2020) 21:5004. doi: 10.3390/ijms21145004. PMID: 32679873 PMC7404130

[116] Yang H Pu S Shu P Wang J Chen Y Yang X . A label-free electrochemical biosensor for sensitive analysis of the PARP-1 activity. Bioelectrochemistry. (2025) 163:108891. doi: 10.1016/j.bioelechem.2024.108891. PMID: 39736194

[117] Yu J Luo L Hu T Cui Y Sun X Gou W . Structure-based design, synthesis, and evaluation of inhibitors with high selectivity for PARP-1 over PARP-2. Eur J Med Chem. (2022) 227:113898. doi: 10.1016/j.ejmech.2021.113898. PMID: 34656898

[118] Wang S Huang J Zeng T Chen Y Xu Y Zhang B . Parps in immune response: Potential targets for cancer immunotherapy. Biochem Pharmacol. (2025) 234:116803. doi: 10.1016/j.bcp.2025.116803. PMID: 39965743

[119] Zhang X Bai X Chen ZJ . Structures and mechanisms in the cGAS-STING innate immunity pathway. Immunity. (2020) 53:43–53. doi: 10.1016/j.immuni.2020.05.013. PMID: 32668227

[120] Hong Z Ma T Liu X Wang C . cGAS‐STING pathway: Post‐translational modifications and functions in sterile inflammatory diseases. FEBS J. (2022) 289:6187–208. doi: 10.1111/febs.16137. PMID: 34310043

[121] Murthy AMV Robinson N Kumar S . Crosstalk between cGAS–STING signaling and cell death. Cell Death Differ. (2020) 27:2989–3003. doi: 10.1038/s41418-020-00624-8. PMID: 32948836 PMC7560597

[122] Yuan M Shi L Liu Y Xiang K Zhang Y Zhou Y . Disulfiram/copper triggers cGAS-STING innate immunity pathway via ROS-induced DNA damage that potentiates antitumor response to PD-1 checkpoint blockade. Int J Biol Sci. (2025) 21:1730–48. doi: 10.7150/ijbs.105575. PMID: 39990655 PMC11844283

[123] Böhi F Hottiger MO . Expanding the perspective on PARP1 and its inhibitors in cancer therapy: From DNA damage repair to immunomodulation. Biomedicines. (2024) 12:1617. doi: 10.3390/biomedicines12071617. PMID: 39062190 PMC11275100

[124] Pantelidou C Sonzogni O De Oliveria Taveira M Mehta AK Kothari A Wang D . PARP inhibitor efficacy depends on CD8+ T-cell recruitment via intratumoral STING pathway activation in BRCA-deficient models of triple-negative breast cancer. Cancer Discov. (2019) 9:722–37. doi: 10.1158/2159-8290.CD-18-1218. PMID: 31015319 PMC6548644

[125] Ding L Kim H-J Wang Q Kearns M Jiang T Ohlson CE . PARP inhibition elicits STING-dependent antitumor immunity in Brca1-deficient ovarian cancer. Cell Rep. (2018) 25:2972–80:e5. doi: 10.1016/j.celrep.2018.11.054. PMID: 30540933 PMC6366450

[126] Li H Li Q Li W Xie L Zhou M Xie J . The role of PARP-1 in host-pathogen interaction and cellular stress responses. Crit Rev Eukaryot Gene Expr. (2015) 25:175–90. doi: 10.1615/CritRevEukaryotGeneExpr.2015013626. PMID: 26080611

[127] Wang C Du M Huang D Huang K Huang K . Inhibition of PARP1 increases IRF-dependent gene transcription in Jurkat cells. Curr Med Sci. (2019) 39:356–62. doi: 10.1007/s11596-019-2043-1. PMID: 31209803

[128] Xiao D Zeng T Zhu W Yu Z-Z Huang W Yi H . ANXA1 promotes tumor immune evasion by binding PARP1 and upregulating Stat3-induced expression of PD-L1 in multiple cancers. Cancer Immunol Res. (2023) 11:1367–83. doi: 10.1158/2326-6066.CIR-22-0896. PMID: 37566399

[129] Ma L Qin N Wan W Song S Hua S Jiang C . TLR9 activation induces immunosuppression and tumorigenesis via PARP1/PD-L1 signaling pathway in oral squamous cell carcinoma. Am J Physiol Cell Physiol. (2024) 326:C362–81. doi: 10.1152/ajpcell.00061.2023. PMID: 38105756

[130] Wang Y Chen H . Protein glycosylation alterations in hepatocellular carcinoma: Function and clinical implications. Oncogene. (2023) 42:1970–9. doi: 10.1038/s41388-023-02702-w. PMID: 37193819 PMC10256610

[131] Agrawal M Agrawal SK Chopra K . Overcoming drug resistance in ovarian cancer through PI3K/AKT signaling inhibitors. Gene. (2025) 948:149352. doi: 10.1016/j.gene.2025.149352. PMID: 39988188

[132] Ji M Shi H Xie Y Zhao Z Li S Chang C . Ubiquitin specific protease 22 promotes cell proliferation and tumor growth of epithelial ovarian cancer through synergy with transforming growth factor β1. Oncol Rep. (2015) 33:133–40. doi: 10.3892/or.2014.3580. PMID: 25369910

[133] Giamougiannis P Martin-Hirsch PL Martin FL . The evolving role of MUC16 (CA125) in the transformation of ovarian cells and the progression of neoplasia. Carcinogenesis. (2021) 42:327–43. doi: 10.1093/carcin/bgab010. PMID: 33608706

[134] Borley J Brown R . Epigenetic mechanisms and therapeutic targets of chemotherapy resistance in epithelial ovarian cancer. Ann Med. (2015) 47:359–69. doi: 10.3109/07853890.2015.1043140. PMID: 26158617

[135] Wieboldt R Sandholzer M Carlini E Lin C-W Börsch A Zingg A . Engagement of sialylated glycans with Siglec receptors on suppressive myeloid cells inhibits anticancer immunity via CCL2. Cell Mol Immunol. (2024) 21:495–509. doi: 10.1038/s41423-024-01142-0. PMID: 38448555 PMC11061307

[136] Bi X Lv X Liu D Guo H Yao G Wang L . METTL3-mediated maturation of miR-126-5p promotes ovarian cancer progression via PTEN-mediated PI3K/Akt/mTOR pathway. Cancer Gene Ther. (2021) 28:335–49. doi: 10.1038/s41417-020-00222-3. PMID: 32939058

[137] Wei B Yang F Yu L Qiu C . Crosstalk between SUMOylation and other post-translational modifications in breast cancer. Cell Mol Biol Lett. (2024) 29:107. doi: 10.1186/s11658-024-00624-3. PMID: 39127633 PMC11316377

[138] Acharya G Mani C Sah N Saamarthy K Young R Reedy MB . CHK1 inhibitor induced PARylation by targeting PARG causes excessive replication and metabolic stress and overcomes chemoresistance in ovarian cancer. Cell Death Discov. (2024) 10:278. doi: 10.1038/s41420-024-02040-0. PMID: 38862485 PMC11166985

[139] Duan X Xing Z Qiao L Qin S Zhao X Gong Y . The role of histone post-translational modifications in cancer and cancer immunity: Functions, mechanisms and therapeutic implications. Front Immunol. (2024) 15:1495221. doi: 10.3389/fimmu.2024.1495221. PMID: 39620228 PMC11604627

[140] Hu Q Shi Y Wang H Bing L Xu Z . Post-translational modifications of immune checkpoints: Unlocking new potentials in cancer immunotherapy. Exp Hematol Oncol. (2025) 14:37. doi: 10.1186/s40164-025-00627-6. PMID: 40087690 PMC11907956

[141] Marchese E Demehri S . Posttranslational protein modifications as gatekeepers of cancer immunogenicity. J Clin Invest. (2024) 134:e180914. doi: 10.1172/JCI180914. PMID: 38747288 PMC11093601

[142] Fulda S Rajalingam K Dikic I . Ubiquitylation in immune disorders and cancer: From molecular mechanisms to therapeutic implications. EMBO Mol Med. (2012) 4:545–56. doi: 10.1002/emmm.201100707. PMID: 22730341 PMC3407942

[143] He Y Song T Ning J Wang Z Yin Z Jiang P . Lactylation in cancer: Mechanisms in tumour biology and therapeutic potentials. Clin Trans Med. (2024) 14:e70070. doi: 10.1002/ctm2.70070. PMID: 39456119 PMC11511673

[144] Srivastava AK Guadagnin G Cappello P Novelli F . Post-translational modifications in tumor-associated antigens as a platform for novel immuno-oncology therapies. Cancers. (2022) 15:138. doi: 10.3390/cancers15010138. PMID: 36612133 PMC9817968

[145] Xu Z Zhang Y Ocansey DKW Wang B Mao F . Glycosylation in cervical cancer: New insights and clinical implications. Front Oncol. (2021) 11:706862. doi: 10.3389/fonc.2021.706862. PMID: 34485140 PMC8415776

[146] Li Z Dong X Liu J Wen T . Decoding aberrant glycosylation in colorectal cancer: From glycosyaltion characterization, expression regulation to potential clinical applications. Biochim Biophys Acta Rev Cancer. (2026) 1881:189513. doi: 10.1016/j.bbcan.2025.189513. PMID: 41371454

[147] Belisle JA Horibata S Jennifer GAA Petrie S Kapur A André S . Identification of Siglec-9 as the receptor for MUC16 on human NK cells, B cells, and monocytes. Mol Cancer. (2010) 9:118. doi: 10.1186/1476-4598-9-118. PMID: 20497550 PMC2890604

[148] Hou R Jiang L Liu D Lin B Hu Z Gao J . Lewis(y) antigen promotes the progression of epithelial ovarian cancer by stimulating MUC1 expression. Int J Mol Med. (2017) 40:293–302. doi: 10.3892/ijmm.2017.3009. PMID: 28586014 PMC5504979

[149] Van Elssen CHMJ Frings PWH Bot FJ Van de Vijver KK Huls MB Meek B . Expression of aberrantly glycosylated mucin-1 in ovarian cancer. Histopathology. (2010) 57:597–606. doi: 10.1111/j.1365-2559.2010.03667.x. PMID: 20955385

[150] Kaufman B Abu-Ahmad M Radinsky O Gharra E Manko T Bhattacharya B . N-glycosylation of PD-L1 modulates the efficacy of immune checkpoint blockades targeting PD-L1 and PD-1. Mol Cancer. (2025) 24:140. doi: 10.1186/s12943-025-02330-w. PMID: 40346531 PMC12065222

[151] Zhu D Xu R Huang X Tang Z Tian Y Zhang J . Deubiquitinating enzyme OTUB1 promotes cancer cell immunosuppression via preventing ER-associated degradation of immune checkpoint protein PD-L1. Cell Death Differ. (2021) 28:1773–89. doi: 10.1038/s41418-020-00700-z. PMID: 33328570 PMC8184985

[152] Xie J Zhang P Liu Y Wu D Ou X Wang M . USP5-mediated PD-L1 deubiquitination regulates immunotherapy efficacy in melanoma. J Transl Med. (2025) 23:778. doi: 10.1186/s12967-025-06812-9. PMID: 40640907 PMC12247207

[153] Madsen CB Petersen C Lavrsen K Harndahl M Buus S Clausen H . Cancer associated aberrant protein O-glycosylation can modify antigen processing and immune response. PloS One. (2012) 7:e50139. doi: 10.1371/journal.pone.0050139. PMID: 23189185 PMC3506546

[154] Stewart N Daly J Drummond-Guy O Krishnamoorthy V Stark JC Riley NM . The glycoimmune checkpoint receptor Siglec-7 interacts with T-cell ligands and regulates T-cell activation. J Biol Chem. (2024) 300:105579. doi: 10.1016/j.jbc.2023.105579. PMID: 38141764 PMC10831161

[155] De Sanctis F Sandri S Ferrarini G Pagliarello I Sartoris S Ugel S . The emerging immunological role of post-translational modifications by reactive nitrogen species in cancer microenvironment. Front Immunol. (2014) 5:69. doi: 10.3389/fimmu.2014.00069. PMID: 24605112 PMC3932549

[156] Piao Y Zhai N Zhang X Zhao W Li M . Post-translational modifications in hepatocellular carcinoma: unlocking new frontiers in immunotherapy. Front Immunol. (2025) 16:1554372. doi: 10.3389/fimmu.2025.1554372. PMID: 40040703 PMC11876159

[157] Sharma S Sarkar O Ghosh R . Exploring the role of unconventional post-translational modifications in cancer diagnostics and therapy. CPPS. (2024) 25:780–96. doi: 10.2174/0113892037274615240528113148. PMID: 38910429

[158] Sun X Xiao C Wang X Wu S Yang Z Sui B . Role of post-translational modifications of Sp1 in cancer: state of the art. Front Cell Dev Biol. (2024) 12:1412461. doi: 10.3389/fcell.2024.1412461. PMID: 39228402 PMC11368732

[159] Huang D Wang J Chen L Jiang W Inuzuka H Simon DK . Targeting the PARylation-dependent ubiquitination signaling pathway for cancer therapies. Biomolecules. (2025) 15:237. doi: 10.3390/biom15020237. PMID: 40001540 PMC11852910

[160] Fischbach A Krüger A Hampp S Assmann G Rank L Hufnagel M . The C-terminal domain of p53 orchestrates the interplay between non-covalent and covalent poly(ADP-ribosyl)ation of p53 by PARP1. Nucleic Acids Res. (2018) 46:804–22. doi: 10.1093/nar/gkx1205. PMID: 29216372 PMC5778597

[161] Ilić N Tao Y Boutros‐Suleiman S Kadali VN Emanuelli A Levy‐Cohen G . SMURF2‐mediated ubiquitin signaling plays an essential role in the regulation of PARP1 PARylating activity, molecular interactions, and functions in mammalian cells. FASEB J. (2021) 35:e21436. doi: 10.1096/fj.202001759R. PMID: 33734501

[162] Lodovichi S Quadri R Sertic S Pellicioli A . PARylation of BRCA1 limits DNA break resection through BRCA2 and EXO1. Cell Rep. (2023) 42:112060. doi: 10.1016/j.celrep.2023.112060. PMID: 36735534

[163] Watson ZL Yamamoto TM McMellen A Kim H Hughes CJ Wheeler LJ . Histone methyltransferases EHMT1 and EHMT2 (GLP/G9A) maintain PARP inhibitor resistance in high-grade serous ovarian carcinoma. Clin Epigenet. (2019) 11:165. doi: 10.1186/s13148-019-0758-2. PMID: 31775874 PMC6882350

[164] Yang F Chen J Liu B Gao G Sebastian M Jeter C . SPINDOC binds PARP1 to facilitate PARylation. Nat Commun. (2021) 12:6362. doi: 10.1038/s41467-021-26588-y. PMID: 34737271 PMC8568969

[165] Konstantinopoulos PA Matulonis UA . PARP inhibitors in ovarian cancer: a trailblazing and transformative journey. Clin Cancer Res. (2018) 24:4062–5. doi: 10.1158/1078-0432.CCR-18-1314. PMID: 29871906

[166] Cook SA Tinker AV . PARP inhibitors and the evolving landscape of ovarian cancer management: a review. BioDrugs. (2019) 33:255–73. doi: 10.1007/s40259-019-00347-4. PMID: 30895466

[167] Kubalanza K Konecny GE . Mechanisms of PARP inhibitor resistance in ovarian cancer. Curr Opin Obstet Gynecol. (2020) 32:36–41. doi: 10.1097/GCO.0000000000000600. PMID: 31815769

[168] Bhatia T Doshi G Godad A . PARP inhibitors in ovarian cancer: mechanisms, resistance, and the promise of combination therapy. Pathol Res Pract. (2024) 263:155617. doi: 10.1016/j.prp.2024.155617. PMID: 39357181

[169] Hou D Liu Z Xu X Liu Q Zhang X Kong B . Increased oxidative stress mediates the antitumor effect of PARP inhibition in ovarian cancer. Redox Biol. (2018) 17:99–111. doi: 10.1016/j.redox.2018.03.016. PMID: 29684820 PMC6006521

[170] Conrad LB Lin KY Nandu T Gibson BA Lea JS Kraus WL . ADP-ribosylation levels and patterns correlate with gene expression and clinical outcomes in ovarian cancers. Mol Cancer Ther. (2020) 19:282–91. doi: 10.1158/1535-7163.MCT-19-0569. PMID: 31594824 PMC7153754

[171] Beretta GL Costantino M Mirra L Pettinari P Perego P . Deubiquitinases in ovarian cancer: role in drug resistance and tumor aggressiveness. Int J Biol Sci. (2024) 20:5208–22. doi: 10.7150/ijbs.100355. PMID: 39430244 PMC11489175

[172] Gasparri ML Besharat ZM Farooqi AA Khalid S Taghavi K Besharat RA . MiRNAs and their interplay with PI3K/AKT/mTOR pathway in ovarian cancer cells: a potential role in platinum resistance. J Cancer Res Clin Oncol. (2018) 144:2313–8. doi: 10.1007/s00432-018-2737-y. PMID: 30109500 PMC11813288

[173] Lund RJ Huhtinen K Salmi J Rantala J Nguyen EV Moulder R . DNA methylation and transcriptome changes associated with cisplatin resistance in ovarian cancer. Sci Rep. (2017) 7:1469. doi: 10.1038/s41598-017-01624-4. PMID: 28473707 PMC5431431

[174] van der Gun BTF de Groote ML Kazemier HG Arendzen AJ Terpstra P Ruiters MHJ . Transcription factors and molecular epigenetic marks underlying EpCAM overexpression in ovarian cancer. Br J Cancer. (2011) 105:312–9. doi: 10.1038/bjc.2011.231. PMID: 21694727 PMC3142811

[175] Min K-J So KA Ouh Y-T Hong J-H Lee J-K . The effects of DNA methylation and epigenetic factors on the expression of CD133 in ovarian cancers. J Ovarian Res. (2012) 5:28. doi: 10.1186/1757-2215-5-28. PMID: 23067401 PMC3489563

[176] Koestler DC Chalise P Cicek MS Cunningham JM Armasu S Larson MC . Integrative genomic analysis identifies epigenetic marks that mediate genetic risk for epithelial ovarian cancer. BMC Med Genomics. (2014) 7:8. doi: 10.1186/1755-8794-7-8. PMID: 24479488 PMC3916313

[177] Yu L . Unraveling role of ubiquitination in drug resistance of gynecological cancer. Am J Cancer Res. (2024) 14:2523–37. doi: 10.62347/WYKZ9784. PMID: 38859858 PMC11162667

[178] Pieterse Z Amaya-Padilla MA Singomat T Binju M Madjid BD Yu Y . Ovarian cancer stem cells and their role in drug resistance. Int J Biochem Cell Biol. (2019) 106:117–26. doi: 10.1016/j.biocel.2018.11.012. PMID: 30508594

[179] Mittica G Ghisoni E Giannone G Genta S Aglietta M Sapino A . PARP inhibitors in ovarian cancer. PRA. (2018) 13:392–410. doi: 10.2174/1574892813666180305165256. PMID: 29512470

[180] Xiao F Wang Z Qiao L Zhang X Wu N Wang J . Application of PARP inhibitors combined with immune checkpoint inhibitors in ovarian cancer. J Transl Med. (2024) 22:778. doi: 10.1186/s12967-024-05583-z. PMID: 39169400 PMC11337781

[181] Rinne N Christie EL Ardasheva A Kwok CH Demchenko N Low C . Targeting the PI3K/AKT/mTOR pathway in epithelial ovarian cancer, therapeutic treatment options for platinum-resistant ovarian cancer. CDR. (2021) 4:573–95. doi: 10.20517/cdr.2021.05. PMID: 35582310 PMC9019160

[182] Turaga SM Hembruff SL Savelieff MG Ghosh A Puri RV Pathak HB . Dual targeting of Aurora Kinase A and poly (ADP-ribose) polymerase as a therapeutic option for patients with ovarian cancer: preclinical evaluations. J Cancer Res Clin Oncol. (2025) 151:124. doi: 10.1007/s00432-025-06152-7. PMID: 40138020 PMC11946953

[183] Guo H . Activation of cGAS confers PARP inhibitor resistance in ovarian cancer via the TBK1-IRF3 axis. Int J Clin Exp Pathol. (2024) 17:429–38. doi: 10.62347/XOPN6908. PMID: 39660334 PMC11626293

[184] Li X Ng ASN Mak VCY Chan KKL Cheung ANY Cheung LWT . Strategic combination therapies for ovarian cancer. CCDT. (2020) 20:573–85. doi: 10.2174/1568009620666200511084007. PMID: 32392113

[185] Veneziani AC Scott C Wakefield MJ Tinker AV Lheureux S . Fighting resistance: post-PARP inhibitor treatment strategies in ovarian cancer. Ther Adv Med Oncol. (2023) 15:17588359231157644. doi: 10.1177/17588359231157644. PMID: 36872947 PMC9983116

[186] Wu L Wang J Li Q Wang D Zhang C Tang J . Fuzuloparib with or without apatinib as maintenance therapy in newly diagnosed, advanced ovarian cancer (FZOCUS‐1): a multicenter, randomized, double‐blind, placebo‐controlled phase 3 trial. CA A Cancer J Clin. (2026) 76:e70042. doi: 10.3322/caac.70042. PMID: 41287969 PMC12645347

[187] Kawczak P Bączek T . Targeting VEGF, PARP, and FRα pathways in ovarian cancer: clinical advances with bevacizumab, rucaparib, and mirvetuximab soravtansine. JCM. (2026) 15:1742. doi: 10.3390/jcm15051742. PMID: 41827159 PMC12986000

[188] Ray-Coquard I Pautier P Pignata S Pérol D González-Martín A Berger R . Olaparib plus bevacizumab as first-line maintenance in ovarian cancer. N Engl J Med. (2019) 381:2416–28. doi: 10.1056/NEJMoa1911361. PMID: 31851799

[189] Shah PD Wethington SL Pagan C Latif N Tanyi J Martin LP . Combination ATR and PARP inhibitor (CAPRI): a phase 2 study of ceralasertib plus olaparib in patients with recurrent, platinum-resistant epithelial ovarian cancer. Gynecol Oncol. (2021) 163:246–53. doi: 10.1016/j.ygyno.2021.08.024. PMID: 34620496 PMC9614917

[190] Wethington SL Shah PD Martin L Tanyi JL Latif N Morgan M . Combination ATR (ceralasertib) and PARP (olaparib) inhibitor (CAPRI) trial in acquired PARP inhibitor–resistant homologous recombination–deficient ovarian cancer. Clin Cancer Res. (2023) 29:2800–7. doi: 10.1158/1078-0432.CCR-22-2444. PMID: 37097611 PMC11934101

[191] Agarwal N Azad AA Carles J Fay AP Matsubara N Szczylik C . Talazoparib plus enzalutamide in men with metastatic castration-resistant prostate cancer: final overall survival results from the randomised, placebo-controlled, phase 3 TALAPRO-2 trial. Lancet. (2025) 406:447–60. doi: 10.1016/S0140-6736(25)00684-1. PMID: 40683290

[192] Mahdi H Hafez N Doroshow D Sohal D Keedy V Do KT . Ceralasertib-mediated ATR inhibition combined with olaparib in advanced cancers harboring DNA damage response and repair alterations (Olaparib combinations). JCO Precis Oncol. (2021) 5:1432–42. doi: 10.1200/PO.20.00439. PMID: 34527850 PMC8437220

[193] Biegała Ł Gajek A Marczak A Rogalska A . PARP inhibitor resistance in ovarian cancer: underlying mechanisms and therapeutic approaches targeting the ATR/CHK1 pathway. Biochim Biophys Acta (BBA) - Rev Cancer. (2021) 1876:188633. doi: 10.1016/j.bbcan.2021.188633. PMID: 34619333

[194] Kulkarni S Seneviratne N Tosun Ç MadhuSudan S . PARP inhibitors in ovarian cancer: mechanisms of resistance and implications to therapy. DNA Repair. (2025) 149:103830. doi: 10.1016/j.dnarep.2025.103830. PMID: 40203475

[195] Zhang L Nadeem L Connor K Xu G . Mechanisms and therapeutic targets of microRNA-associated chemoresistance in epithelial ovarian cancer. CCDT. (2016) 16:429–41. doi: 10.2174/1568009616666160404121105. PMID: 27040353

[196] Ponton-Almodovar A Sanderson S Rattan R Bernard JJ Horibata S . Ovarian tumor microenvironment contributes to tumor progression and chemoresistance. Cancer Drug Resist. (2024) 7:53. doi: 10.20517/cdr.2024.111. PMID: 39802952 PMC11724355

[197] Ma Y-T Li C Shen Y You W-H Han M-X Mu Y-F . Mechanisms of the JNK/p38 MAPK signaling pathway in drug resistance in ovarian cancer. Front Oncol. (2025) 15:1533352. doi: 10.3389/fonc.2025.1533352. PMID: 40352594 PMC12063130

[198] Miller EM Samec TM Alexander-Bryant AA . Nanoparticle delivery systems to combat drug resistance in ovarian cancer. Nanomed: Nanotechnol Biol Med. (2021) 31:102309. doi: 10.1016/j.nano.2020.102309. PMID: 32992019

[199] Kapper C Oppelt P Arbeithuber B Gyunesh AA Vilusic I Stelzl P . Targeting ferroptosis in ovarian cancer: novel strategies to overcome chemotherapy resistance. Life Sci. (2024) 349:122720. doi: 10.1016/j.lfs.2024.122720. PMID: 38762066

[200] Kumbhar R Vidal-Eychenié S Kontopoulos D-G Larroque M Larroque C Basbous J . Recruitment of ubiquitin-activating enzyme UBA1 to DNA by poly(ADP-ribose) promotes ATR signalling. Life Sci Alliance. (2018) 1:e201800096. doi: 10.26508/lsa.201800096. PMID: 30456359 PMC6238597

[201] Hamilton EP Falchook GS Wang JS Fu S Oza AM Imedio ER . Adavosertib in combination with olaparib in patients with refractory solid tumors: an open-label, dose-finding, and dose-expansion phase Ib trial. Target Oncol. (2024) 19:879–92. doi: 10.1007/s11523-024-01102-8. PMID: 39487373 PMC11557630

[202] Lheureux S Cristea MC Bruce JP Garg S Cabanero M Mantia-Smaldone G . Adavosertib plus gemcitabine for platinum-resistant or platinum-refractory recurrent ovarian cancer: a double-blind, randomised, placebo-controlled, phase 2 trial. Lancet. (2021) 397:281–92. doi: 10.1016/S0140-6736(20)32554-X. PMID: 33485453 PMC10792546

[203] Fu S Yao S Yuan Y Previs RA Elias AD Carvajal RD . Multicenter phase II trial of the WEE1 inhibitor adavosertib in refractory solid tumors harboring CCNE1 amplification. J Clin Oncol. (2023) 41:1725–34. doi: 10.1200/JCO.22.00830. PMID: 36469840 PMC10489509

[204] Nguyen NT Raetz A Montoya D Schilling V Tong C Brooks RA . Targeting RAS-ERK pathway alterations with MEK inhibitors to improve chemosensitivity in high grade serous ovarian cancers. Gynecol Oncol. (2023) 178:69–79. doi: 10.1016/j.ygyno.2023.09.014. PMID: 37806229

[205] Freyer G Floquet A Tredan O Carrot A Langlois-Jacques C Lopez J . Bevacizumab, olaparib, and durvalumab in patients with relapsed ovarian cancer: a phase II clinical trial from the GINECO group. Nat Commun. (2024) 15:1985. doi: 10.1038/s41467-024-45974-w. PMID: 38443333 PMC10914754

[206] Liu J Jiao X Mu W Li H Xia Y Wu Y . Mitigating T cell DNA damage during PARP inhibitor treatment enhances antitumor efficacy. Sci Transl Med. (2025) 17:eadr5861. doi: 10.1126/scitranslmed.adr5861. PMID: 40333991

[207] Sen T Rodriguez BL Chen L Corte CMD Morikawa N Fujimoto J . Targeting DNA damage response promotes antitumor immunity through STING-mediated T-cell activation in small cell lung cancer. Cancer Discov. (2019) 9:646–61. doi: 10.1158/2159-8290.CD-18-1020. PMID: 30777870 PMC6563834

[208] Jiao X Liu J Wu Y Zhong Q Zhu L Wang L . Sequential treatment with PARPi and WEE1i enhances antitumor immune responses in preclinical models of ovarian cancer. Sci Transl Med. (2025) 17:eadu6989. doi: 10.1126/scitranslmed.adu6989. PMID: 40802736

[209] Kim H Xu H George E Hallberg D Kumar S Jagannathan V . Combining PARP with ATR inhibition overcomes PARP inhibitor and platinum resistance in ovarian cancer models. Nat Commun. (2020) 11:3726. doi: 10.1038/s41467-020-17127-2. PMID: 32709856 PMC7381609

[210] Zhang X Yao J Li X Niu N Liu Y Hajek RA . Targeting polyploid giant cancer cells potentiates a therapeutic response and overcomes resistance to PARP inhibitors in ovarian cancer. Sci Adv. (2023) 9:eadf7195. doi: 10.1126/sciadv.adf7195. PMID: 37478190 PMC10361597

[211] Li T Wang X Qin S Chen B Yi M Zhou J . Targeting PARP for the optimal immunotherapy efficiency in gynecologic Malignancies. Biomed Pharmacother. (2023) 162:114712. doi: 10.1016/j.biopha.2023.114712. PMID: 37075667

[212] Muñoz-Galván S Carnero A . Targeting cancer stem cells to overcome therapy resistance in ovarian cancer. Cells. (2020) 9:1402. doi: 10.3390/cells9061402. PMID: 32512891 PMC7349391

[213] Zhang Y Dong Y Fu H Huang H Wu Z Zhao M . Multifunctional tumor-targeted PLGA nanoparticles delivering Pt(IV)/siBIRC5 for US/MRI imaging and overcoming ovarian cancer resistance. Biomaterials. (2021) 269:120478. doi: 10.1016/j.biomaterials.2020.120478. PMID: 33213862

[214] Liu L Liu P Liang Z Li R Shen M Xu H . Poly (ADP-ribose) polymerase inhibitors combined with other small-molecular compounds for the treatment of ovarian cancer. Anticancer Drugs. (2019) 30:554–61. doi: 10.1097/CAD.0000000000000793. PMID: 30998513

[215] Garlisi B Lauks S Aitken C Ogilvie LM Lockington C Petrik D . The complex tumor microenvironment in ovarian cancer: therapeutic challenges and opportunities. Curr Oncol. (2024) 31:3826–44. doi: 10.3390/curroncol31070283. PMID: 39057155 PMC11275383

[216] Vorderbruggen M Velázquez-Martínez CA Natarajan A Karpf AR . PROTACs in ovarian cancer: current advancements and future perspectives. IJMS. (2024) 25:5067. doi: 10.3390/ijms25105067. PMID: 38791105 PMC11121112

[217] Ibrahim S Umer Khan M Khurram I Rehman R Rauf A Ahmad Z . Navigating PROTACs in cancer therapy: advancements, challenges, and future horizons. Food Sci Nutr. (2025) 13:e70011. doi: 10.1002/fsn3.70011. PMID: 39898116 PMC11786021

[218] Wang Y-Y Choi M-J Kim J-H Choi J-H . Enhanced expression of TRIM46 in ovarian cancer cells induced by tumor-associated macrophages promotes invasion via the Wnt/β-catenin pathway. Cells. (2025) 14:214. doi: 10.3390/cells14030214. PMID: 39937005 PMC11817100

[219] Asl ER Sarabandi S Shademan B Dalvandi K sheikhansari G Nourazarian A . MicroRNA targeting: a novel therapeutic intervention for ovarian cancer. Biochem Biophys Rep. (2023) 35:101519. doi: 10.1016/j.bbrep.2023.101519. PMID: 37521375 PMC10382632

[220] Saman S Srivastava N Yasir M Chauhan I . A comprehensive review on current treatments and challenges involved in the treatment of ovarian cancer. CCDT. (2024) 24:142–66. doi: 10.2174/1568009623666230811093139. PMID: 37642226

[221] Kim H-K Cheong H Kim M-Y Jin H-E . Therapeutic targeting in ovarian cancer: nano-enhanced CRISPR/Cas9 gene editing and drug combination therapy. IJN. (2025) 20:3907–31. doi: 10.2147/IJN.S507688. PMID: 40191042 PMC11970428

[222] Zhou JX Feng LJ Zhang X . Risk of severe hematologic toxicities in cancer patients treated with PARP inhibitors: a meta-analysis of randomized controlled trials. Drug Des Devel Ther. (2017) 11:3009–17. doi: 10.2147/DDDT.S147726. PMID: 29075104 PMC5648323

[223] Shu Y Ding Y He X Liu Y Wu P Zhang Q . Hematological toxicities in PARP inhibitors: a real-world study using FDA adverse event reporting system (FAERS) database. Cancer Med. (2023) 12:3365–75. doi: 10.1002/cam4.5062. PMID: 35871395 PMC9939145

[224] Smith HL Willmore E Prendergast L Curtin NJ . ATR, CHK1 and WEE1 inhibitors cause homologous recombination repair deficiency to induce synthetic lethality with PARP inhibitors. Br J Cancer. (2024) 131:905–17. doi: 10.1038/s41416-024-02745-0. PMID: 38965423 PMC11369084

[225] Kim H George E Ragland R Rafail S Zhang R Krepler C . Targeting the ATR/CHK1 axis with PARP inhibition results in tumor regression in BRCA-mutant ovarian cancer models. Clin Cancer Res. (2017) 23:3097–108. doi: 10.1158/1078-0432.CCR-16-2273. PMID: 27993965 PMC5474193

[226] Ramos-Casals M Sisó-Almirall A . Immune-related adverse events of immune checkpoint inhibitors. Ann Intern Med. (2024) 177:ITC17–32. doi: 10.7326/AITC202402200. PMID: 38346306

[227] Das S Johnson DB . Immune-related adverse events and anti-tumor efficacy of immune checkpoint inhibitors. J Immunother Cancer. (2019) 7:306. doi: 10.1186/s40425-019-0805-8. PMID: 31730012 PMC6858629

[228] Lin KK Harrell MI Oza AM Oaknin A Ray-Coquard I Tinker AV . BRCA reversion mutations in circulating tumor DNA predict primary and acquired resistance to the PARP inhibitor rucaparib in high-grade ovarian carcinoma. Cancer Discov. (2019) 9:210–9. doi: 10.1158/2159-8290.CD-18-0715. PMID: 30425037

[229] Haynes B Murai J Lee J-M . Restored replication fork stabilization, a mechanism of PARP inhibitor resistance, can be overcome by cell cycle checkpoint inhibition. Cancer Treat Rev. (2018) 71:1–7. doi: 10.1016/j.ctrv.2018.09.003. PMID: 30269007 PMC7429716

[230] Yazinski SA Comaills V Buisson R Genois M-M Nguyen HD Ho CK . ATR inhibition disrupts rewired homologous recombination and fork protection pathways in PARP inhibitor-resistant BRCA-deficient cancer cells. Genes Dev. (2017) 31:318–32. doi: 10.1101/gad.290957.116. PMID: 28242626 PMC5358727

[231] Song K-H Kim J-H Lee Y-H Bae HC Lee H-J Woo SR . Mitochondrial reprogramming via ATP5H loss promotes multimodal cancer therapy resistance. J Clin Invest. (2018) 128:4098–114. doi: 10.1172/JCI96804. PMID: 30124467 PMC6118592

[232] Hirpara J Eu JQ Tan JKM Wong AL Clement M-V Kong LR . Metabolic reprogramming of oncogene-addicted cancer cells to OXPHOS as a mechanism of drug resistance. Redox Biol. (2019) 25:101076. doi: 10.1016/j.redox.2018.101076. PMID: 30642723 PMC6859574

[233] Risnayanti C Jang Y-S Lee J Ahn HJ . PLGA nanoparticles co-delivering MDR1 and BCL2 siRNA for overcoming resistance of paclitaxel and cisplatin in recurrent or advanced ovarian cancer. Sci Rep. (2018) 8:7498. doi: 10.1038/s41598-018-25930-7. PMID: 29760419 PMC5951813

[234] Geethadevi A Ku Z Tsaih S-W Parashar D Kadamberi IP Xiong W . Blocking Oncostatin M receptor abrogates STAT3 mediated integrin signaling and overcomes chemoresistance in ovarian cancer. NPJ Precis Onc. (2024) 8:127. doi: 10.1038/s41698-024-00593-y. PMID: 38839865 PMC11153533

[235] Bi R Chen L Huang M Qiao Z Li Z Fan G . Emerging strategies to overcome PARP inhibitors’ resistance in ovarian cancer. Biochim Biophys Acta (BBA) - Rev Cancer. (2024) 1879:189221. doi: 10.1016/j.bbcan.2024.189221. PMID: 39571765

[236] Marolt N Pavlič R Kreft T Gjorgoska M Rižner TL . Targeting estrogen metabolism in high-grade serous ovarian cancer shows promise to overcome platinum resistance. Biomed Pharmacother. (2024) 177:117069. doi: 10.1016/j.biopha.2024.117069. PMID: 38968802

[237] Ai Z Lu Y Qiu S Fan Z . Overcoming cisplatin resistance of ovarian cancer cells by targeting HIF-1-regulated cancer metabolism. Cancer Lett. (2016) 373:36–44. doi: 10.1016/j.canlet.2016.01.009. PMID: 26801746 PMC4769873

[238] Jung E Koh D Lim Y Shin SY Lee YH . Overcoming multidrug resistance by activating unfolded protein response of the endoplasmic reticulum in cisplatin-resistant A2780/CisR ovarian cancer cells. BMB Rep. (2020) 53:88–93. doi: 10.5483/BMBRep.2020.53.2.108. PMID: 31401981 PMC7061211

[239] Ma Y Xue F Pei Z Zhao Y . Constructing a co-culture model of cancer-associated fibroblasts and ovarian cancer organoids and studying mechanisms of drug resistance. Exp Cell Res. (2025) 450:114656. doi: 10.1016/j.yexcr.2025.114656. PMID: 40562199

[240] Wang L Wang X Zhu X Zhong L Jiang Q Wang Y . Drug resistance in ovarian cancer: from mechanism to clinical trial. Mol Cancer. (2024) 23:66. doi: 10.1186/s12943-024-01967-3. PMID: 38539161 PMC10976737

[241] Khan MA Vikramdeo KS Sudan SK Singh S Wilhite A Dasgupta S . Platinum-resistant ovarian cancer: from drug resistance mechanisms to liquid biopsy-based biomarkers for disease management. Semin Cancer Biol. (2021) 77:99–109. doi: 10.1016/j.semcancer.2021.08.005. PMID: 34418576 PMC8665066

[242] Motohara T Katabuchi H . Ovarian cancer stemness: Biological and clinical implications for metastasis and chemotherapy resistance. Cancers. (2019) 11:907. doi: 10.3390/cancers11070907. PMID: 31261739 PMC6678827

